# Brazilian guidelines for the diagnosis and treatment of candidemia and invasive candidiasis in adults and children

**DOI:** 10.1016/j.bjid.2026.105946

**Published:** 2026-07-21

**Authors:** Arnaldo L. Colombo, Marcello M.C. Magri, Fabianne Carlesse, Diego R. Falci, Marcia Garnica, Daniel W.C.L. Santos, Flavio Queiroz-Telles, Alessandro C. Pasqualotto

**Affiliations:** a Universidade Federal de São Paulo (UNIFESP), Instituto de Resistência Antimicrobiana de São Paulo (ARIES), São Paulo, SP, Brazil; b Universidade Federal de São Paulo (UNIFESP), São Paulo, SP, Brazil; c The Latin American Center for Medical Mycology ‒ CMM LATAM, Brazil; d Universidade de São Paulo, Faculdade de Medicina, Hospital das Clínicas, Divisão de Clínica de Moléstias Infecciosas e Parasitárias, São Paulo, SP, Brazil; e Centro Universitário Faculdade de Medicina do ABC (FMABC), Disciplina de Infectologia, Santo André, SP, Brasil; f Grupo de Apoio ao Adolescente e Criança com Câncer (GRAACC), Instituto de Oncologia Pediátrica, São Paulo, SP, Brazil; g Hospital de Clínicas de Porto Alegre, Porto Alegre, RS, Brazil; h Pontifícia Universidade Católica do Rio Grande do Sul, Porto Alegre, RS, Brazil; i Universidade Federal do Rio de Janeiro, Rio de Janeiro, RJ, Brazil; j IDOR ‒ Instituto D'Or de Pesquisa e Ensino, Rede D’Or, São Luís, MA, Brazil; k Hospital Universitário da Universidade Federal do Maranhão, São Luís, MA, Brazil; l Universidade Federal do Parana, Departamento de Saúde Pública, Curitiba, Brazil; m Universidade Federal de Ciências da Saúde de Porto Alegre, Porto Alegre, RS, Brazil; n Santa Casa de Porto Alegre, Porto Alegre, RS, Brazil; o University of Minnesota, Minneapolis, MN, USA

**Keywords:** Candidemia, Invasive candidiasis, Antifungal therapy, Echinocandins, Clinical guidelines, Brazil

## Abstract

**Background:**

Invasive candidiasis, including candidemia and deep-seated *Candida* infections, remains a major cause of morbidity and mortality in hospitalized patients worldwide, particularly in low- and middle-income countries. In Brazil, candidemia is associated with persistently high mortality rates, driven by late diagnosis, limited access to antifungal agents, and heterogeneous diagnostic capacity. The last national guideline issued by the Brazilian Society of Infectious Diseases (SBI) was published in 2013, and substantial advances have occurred since then in epidemiology, diagnostics, and antifungal therapy.

**Objectives:**

To provide an updated, evidence-based Brazilian guideline for the diagnosis and treatment of candidemia and invasive candidiasis in adults and children, incorporating contemporary scientific data and addressing the specific epidemiological, diagnostic, and therapeutic realities of Brazil.

**Methods:**

This guideline was developed by the SBI Mycology Committee through a structured consensus process. Clinical questions were formulated using the PICO framework, followed by systematic literature searches of PubMed-indexed studies and international guidelines. Recommendations were contextualized according to Brazilian epidemiology, laboratory infrastructure, and access to antifungal therapies.

**Results:**

Key updates include revised epidemiological data from Brazilian cohorts, updated recommendations for blood culture strategies and non-culture-based diagnostics, refined indications for echocardiography and fundoscopy, and updated therapeutic algorithms. Novel antifungal agents, including long acting echinocandins such as rezafungin, are discussed. Emphasis is placed on early echinocandin therapy, timely source control, antifungal stewardship, and infectious diseases consultation.

**Conclusions:**

This updated Brazilian guideline reflects current scientific evidence while addressing national healthcare realities, aiming to standardize care, improve outcomes, and reduce mortality associated with invasive candidiasis in Brazil.

## Introduction

*Candida albicans, C. tropicalis* and *C. parapsilosis* complex may be part of the normal human microbiota and were all incorporated to the Fungal Pathogen Priority List recently published by World Health Organization (WHO-FPPL) as part of a global effort to systematically prioritize fungal pathogens, considering their unmet research and development needs as well as their impact in public health. *Candida auris* (now *Candidozyma auris*) and *Candida glabrata* (now *Nakaseomyces glabratus*) were both included in the WHO-FPPL considering their capacity to cause invasive infections and to develop multidrug antifungal resistance. All these mentioned species are considered to be the most common pathogens causing Invasive Candidiasis (IC) in tertiary care hospitals from different continents.^1^, ^2^, ^3^

IC is the most common hospital acquired Invasive Fungal Disease (IFD) worldwide and involve two main different clinical entities: (i) Fungemia due to *Candida* species is typically documented in patients with degenerative and/or neoplastic diseases who undergo prolonged hospitalizations, require intensive care support, are exposed to multiple invasive medical procedures, and receive broad-spectrum antibiotic therapy and (ii) Intra-Abdominal Candidiasis (IAC) most commonly presents as fungal peritonitis, pancreatitis, and/or intra-abdominal abscesses, resulting from *Candida* overgrowth within the abdominal cavity, typically following complicated Gastrointestinal (GI) surgery, gastrointestinal perforation, or necrotizing pancreatitis. Less than 20% of IAC will develop a positive blood culture and up to 40% of patients with secondary and tertiary peritonitis may develop IAC.^2^^,^^4^, ^5^, ^6^ Of note, according to data generated by a multicenter study (EUCANDICU) conducted in 23 Intensive Care Units (ICUs) across nine European countries where 570 episodes of IC were investigated, IAC responds for 29% of all episodes of IC documented in ICU.^6^

The global burden of IC was recently simulated by study conducted by Denning et al. where the authors reviewed data of 85 papers addressing population at risk on individual country and global disease burden to estimate annual incidences of IFDs for 2019‒2021. According to the authors, epidemiological data collected along 85 different nations suggest that there are 1,565,000 people developing *Candida* bloodstream infection or IC each year globally.^7^

Besides being a leading cause of hospital-acquired fungal infection, invasive infections due to *Candida* are associated with high morbidity and mortality rates worldwide.^8^ In order to estimate the attributable mortality of candidemia, a recent paper conducted by Mazi et al. evaluated 626 adult patients with *Candida* bloodstream infection that were matched with 6,269 control patients with similar risk-conditions but without IC. The 90-day associated mortality was 42.4% (269 patients) for *Candida* bloodstream infection cases and 17.1% (1,083 patients) for controls. Following propensity score-matching, the attributable risk difference for 90-day mortality was 28.4% with Hazard Ratio (HR) of 2.12 (95% Confidence Interval [95% CI 1.98‒2.25], p < 0.001).^9^

## Methodology used to develop this guideline

The methodology for the development of the Brazilian consensus on candidemia and IC was coordinated by the Brazilian Society of Infectious Diseases (SBI), which delegated the task to its Mycology Committee. A working group was assembled to draft the consensus document. The group first formulated specific clinical questions to be addressed, covering multiple domains including epidemiology ‒ with emphasis on national data ‒ to include diagnosis, treatment, prevention, and access to antifungal agents.

Each topic was assigned to individual authors, who conducted PubMed search strategies based on the PICO (Population, Intervention, Comparison, Outcome) framework. Authors were asked to present a theoretical rationale for each question, followed by evidence-based recommendations, with careful consideration of the Brazilian context. Multiple online meetings were held to coordinate efforts and discuss methodological strategies, and a face-to-face meeting took place during the ISHAM Congress in Brazil. After drafting the full text, all authors reviewed the final version and contributed comments and revisions to ensure consensus and accuracy. Table 1 shows the grading system used to evaluate strength of recommendation and quality of evidence for this guideline.

**Table 1:** Evidence-based rating system used in this guideline.

Strength of the recommendation	Definition
A	Should always be offered.
B	Should generally be offered.
C	Optional. Evidence for efficacy is insufficient to support a recommendation for or against, or efficacy might not outweigh adverse consequences, or cost of the approach.
D	Should generally not be offered. Moderate evidence for lack of efficacy or for adverse outcome supports a recommendation against use.
E	Should never be offered. Good evidence for lack of efficacy or for adverse outcome supports a recommendation against use.
**Quality of evidence supporting the recommendation**	**Definition**
I	Evidence from at least one properly randomized, controlled trial.
II	Evidence for at least one well-designed clinical trial without randomization, from cohort or case-controlled analytic studies (preferable from more than one center) or from multiple time-series or dramatic results from uncontrolled experiments.
III	Evidence from opinions of respected authorities based on clinical experience, descriptive.

Adapted from Kish MA; Infectious Diseases Society of America. Guide to development of practice guidelines. Clin Infect Dis. 2001; 32: 851-4.

To facilitate clinicians’ understanding, the species names of *Candida* have been retained as traditionally used, with the updated nomenclature adopted in the literature provided in parentheses at the first mention in the text. Changes in fungal species nomenclature have been driven primarily by advances in genetic sequencing studies;^10^^,^^11^ however, this is an evolving field, and such changes may cause considerable confusion for clinicians who are not specialists in mycology. Therefore, we have opted to retain the original names in the text.

## Epidemiology of candidemia in Brazil

### What is the incidence of candidemia in Brazil?

Incidence rates of IC are strongly influenced by geography, economic inequalities, standards of care and risk population evaluated.^7^^,^^12^ There is a shortage of population-based studies conducted in Brazil, but several laboratory-based surveys of candidemia published along the last 4-years suggest that the incidence rates of candidemia in Brazil varies from 1.0‒3.4 cases per 1,000 admissions in tertiary care hospitals.^13^, ^14^, ^15^

Different authors have reported a substantial increase in the number of candidemia that were documented during the Coronavirus Disease 2019 (COVID-19) pandemic in ICUs globally.^13^^,^^16^ In Brazil, Gieburowsk et al. evaluated the impact of COVID-19 pandemic in the incidence rates of candidemia in a tertiary care hospital from Campinas. The authors found an incidence of IC of 3.4 cases per 1,000 admissions in the pre-pandemic period, and 4.5 cases per 1,000 hospital admissions in the pandemic period. Specially among ICU patients, incidence rates of IC increased from 19.5 cases per 1,000 admissions (pre pandemia) to 21.4 cases per 1,000 admissions along the COVID-19 pandemic period.^15^ Substantial increments in the incidence rates of candidemia during COVID-19 pandemic were also reported by other authors in Brazil.^13^^,^^17^

### What are the underlying conditions and risk factors for candidemia in Brazil?

Recently, Thomas-Rüddel et al. (2022) conducted a meta-analysis including a total of 34 studies addressing risk factors for invasive *Candida* infections in critically ill patients. The authors extracted the raw and adjusted Odds Ratio (OR) for each risk factor associated with invasive *Candida* infection. Based on data generated in this study, the risk factors associated with the highest risk for invasive *Candida* infections were broad-spectrum antibiotics (OR=5.6; 95% CI 3.6-8.8), blood transfusion (OR = 4.9; 95% CI 1.5‒16.3), *Candida* colonization (OR = 4.7; 95% CI 1.6‒14.3), Central Venous Catheter (CVC) use (OR = 4.7; 95% CI 2.7‒8.1), and parenteral nutrition (OR = 4.6; 95% CI 3.3‒6.3).^18^

As expected, same risk conditions are associated with episodes of candidemia in Brazilian tertiary care hospitals. Table 2 summarizes the risk conditions associated with candidemia based on data collected along 3 large retrospective surveys of candidemia documented in Brazil.^19^, ^20^, ^21^

**Table 2:** Demographics and risk conditions associated with candidemia in large Brazilian cohort studies.

Variables	Guimaraes (2012)^21^	Agnelli (2022)^19^	Araujo (2024)^20^
Number of patients (number of centers)	987 (14)	397 (5)	115 (1)
**Demographics**			
Age (Median)	57	66	55
Male prevalence (%)	57	57	59
Time (days) from hospital admission to candidemia (Median)	19	NI	21
**Comorbidities (%)**			
Diabetes	15	21	26
Cardiovascular diseases	12	20	11
Kidney failure	16	43	28
Liver failure	5	12	NI
Solid cancer	15	29	NI
Hematologic malignancies	17	7	NI
**Risk factors (%)**			
Corticosteroids	38	42	49.5
Antibiotics	92	79	90
Central venous catheter	64	83	95
Surgery/abdominal surgery	42/21	NI/27	52/NI
Parenteral nutrition	35	20.4	20.9

NI, Data not informed by the authors.

### Etiology of candidemia in Brazilian medical centers: what are the trends in species distribution and antifungal resistance?

The taxonomy of medically significant yeasts, particularly within the order Saccharomycetales, continues to undergo profound reclassification due to advancements in molecular taxonomy. Formerly classified under the genus *Candida*, many species implicated in life-threatening invasive infections have been reclassified due to their evolutionary relationships elucidated by genetic sequencing methods.^22^

Currently, there are at least 15 distinct *Candida* species that may cause human disease, but over 90% of invasive disease is caused by the following 6 species: *C. albicans, C. parapsilosis* complex, *C. tropicalis, C. glabrata, C. krusei* (now *Pichia kudriavzevii)* and *C. auris*.^23^^,^^24^

In Brazil, a recent paper from Hamburguer et al. (2024) reviewed the etiology of 2,306 episodes of candidemia that were reported in 16 articles published between 2017 and 2023 (Table 3). *C. albicans* remains the most common species causing candidemia in all Brazilian medical centers, but there was a noticeable shift towards non-*C. albicans Candida* species which often have lower susceptibility to antifungals.^2^^,^^8^^,^^25^ In most Brazilian studies, non-*C. albicans Candida* species responded for more than 50% of all episodes of IC. The 3 main non-*C. albicans Candida* species associated to fungemia were *C. parapsilosis* complex (11%‒37%), *C. tropicalis* (2%‒28%), and *C. glabrata* (2%‒22%).^25^

**Table 3:** Species distribution of yeast isolates causing fungemia in 16 studies conducted in Brazilian medical centers (adapted from Burguer et al, 2024).

Study (year)	Number of Candida-BSI	*C. albicans*	*C. parapsilosis* complex	*C. tropicalis*	*Nakaseomyces glabratus* (former *C. glabrata*)	*Pichia kudriavzevii* (former *C. krusei*)	Other *Candida* species^a^
Motta et al. (2017)	65	24 (37%)	20 (31%)	5 (8%)	2 (3%)	2 (3%)	12 (18%)
Canela et al. (2017)	79	35 (44%)	14 (18%)	15 (19%)	15 (19%)	NC	NC
Braga et al. (2018)	331	124 (38%)	61 (18%)	93 (28%)	23 (7%)	6 (2%)	24 (7%)
Marins et al. (2018)	113	42 (37%)	26 (23%)	13 (12%)	25 (22%)	5 (4%)	2 (2%)
Breda et al. (2018)	121	63 (52%)	13 (11%)	18 (15%)	18 (15%)	6 (5%)	3 (2%)
Melo et al. (2019)	70	24 (34%)	18 (26%)	18 (26%)	7 (10%)	1 (1%)	2 (3%)
Medeiros et al. (2019)	51	18 (35%)	11 (22%)	14 (27%)	6 (12%)	0 (0%)	2 (4%)
Rodrigues et al. (2019)	94	27 (29%)	33 (35%)	22 (23%)	2 (2%)	2 (2%)	8 (9%)
Silva et al. (2020)	86	32 (37%)	27 (31%)	15 (17%)	2 (2%)	2 (2%)	8 (9%)
Alves et al. (2020)	341	154 (45%)	58 (17%)	75 (22%)	30 (9%)	14 (4%)	10 (3%)
Oliveira et al. (2021)	100	49 (49%)	25 (25%)	15 (15%)	4 (4%)	3 (3%)	4 (4%)
Nucci et al. (2021)	41	18 (44%)	7 (17%)	10 (24%)	4 (10%)	0 (0%)	2 (5%)
Silva et al. (2021)	28	10 (36%)	4 (14%)	2 (7%)	1 (4%)	0 (0%)	11 (39%)
Rodrigues et al. (2021)	144	42 (29%)	53 (37%)	21 (15%)	14 (10%)	4 (3%)	10 (7%)
Agnelli et al. (2023)	597	243 (41%)	131 (22%)	112 (19%)	81 (14%)	25 (4%)	5 (1%)
Silva et al. (2023)	44	14 (32%)	17 (39%)	1 (2%)	2 (5%)	0 (0%)	10 (23%)
**Total**	**2,305**	**919 (40%)**	**518 (22%)**	**449 (20%)**	**236 (10%)**	**70 (3%)**	**113 (5%)**

NC, data Not Collected.

a Other *Candida* species: Includes *Meyerozyma guilliermondii* (37), *Candida haemulonii complex* (18), *Clavispora lusitaniae* (15), *Wickerhamomyces anomalus* (14), *Debaryomyces hansenii* (12), *Kluyveromyces marxianus* (6), and others with fewer occurrences. The total percentage for “Other *Candida* species” is approximately 5% of the aggregate isolates. (Adapted from Hamburger et al., 2024).

In regard to antifungal resistance, besides the substantial increment of episodes of fungemia due to *C. glabrata*, that now represents 10%‒15% of all episodes of fungemia in most tertiary care hospitals in Brazil,^26^ there is a growing concern about the emergence of outbreaks of candidemia due to *C. parapsilosis* isolates resistant to fluconazole, and a trend to see more and more cases of invasive infections due to fluconazole non-susceptible *C. tropicalis* isolates. *Candida* resistance to echinocandins and amphotericin B remains rare in our country.^25^

*C. auris* had not been reported in Brazil until recently.^27^ However, in December 2020, this potentially multi-resistant yeast was isolated in critically ill patients admitted in an ICU for COVID-19 patients, in Salvador.^28^ The outbreak was successfully controlled as new ones reported in Recife, Olinda, Natal, Campinas, Rio de Janeiro, Belo Horizonte and São Paulo. So far, *C. auris* dissemination in Brazil has been successfully controlled and mitigated by a National Taskforce that was created by ANVISA and Brazilian Ministry of Health. However, it should be noted that many Brazilian centers lack adequate capacity to identify *C. auris* at the species level using MALDI-TOF,^29^ meaning that the true magnitude of the problem may be underestimated.

### What are the historical trends of mortality rates of candidemia in Brazilian medical centers?

Mortality rates of candidemia in Brazil are usually higher than those documented in USA and European countries and have not changed substantially along the last decades.^19^, ^20^, ^21^^,^^30^, ^31^, ^32^

A recent paper by Almeida et al. (2025) addressing the main clinical characteristics and outcomes of 314 patients with candidemia admitted in ICUs of 11 medical centers from Brazil across two periods of time, 2010‒2012 vs. 2017‒2018, found 30-days crude mortality rates of 58.8% and 62.6%, respectively (p = 0.721).^32^ Indeed, same results were obtained by Agnelli *et al*. investigating the natural history of 616 episodes of candidemia documented in tertiary care hospitals (ICU and non-ICU patients) in Brazil along two periods: 2010‒2011 (Period I) vs. 2017‒2018 (Period II). Unfortunately, no improvements in mortality rates at 14-days (123 [33.6%] vs. 93 [37.7%], p = 0.343) or at 30-days (188 [51.4%] vs. 120 [48.6%], p = 0.511) were observed.^19^

It is noteworthy that mortality rates of candidemia in Brazilian medical centers are higher than those documented in European countries. Agnelli et al. compared the clinical management of candidemia diagnosed in five tertiary hospitals from Brazil and Spain between 2010‒2018. Overall, 720 patients with candidemia were included in this analysis, being 323 from Spain. Mortality rates were higher in Brazil at 14-days (35.8% vs. 20.1%, p < 0.001), and at 30-days (51.9% vs. 31.6%, p < 0.001) after diagnosis of candidemia. In the same context, a multicenter study investigating mortality rates of candidemia in kidney transplant recipients found 14-days all-cause mortality rates substantially higher in Brazil when compared to patients from Spain and Italy (41% vs. 11%, p = 0.016).^30^

Factors associated with increment in mortality rates of candidemia in Brazilian medical centers are mostly related to late diagnosis, limited access to echinocandins as first line therapy and inadequate source control.^19^^,^^30^, ^31^, ^32^

## Diagnosis

### What culture media can be used to maximize *Candida* spp. detection?

Blood cultures remain the cornerstone for diagnosing candidemia in Brazil and Latin America, but their turnaround is inherently slow, with a median of two to three days to positivity and little additional benefit from incubation beyond five days. The performance of culture varies with the system and bottle type employed.^33^^,^^34^ Fungus-specific bottles, such as Mycosis IC/F, have shown faster detection of *C. albicans* and increased recovery of yeasts in cases with concomitant bacteremia. In contrast, conventional non-selective bottles, such as BacT/Alert FA, may provide superior recovery when patients are receiving antifungal therapy. Despite these observations, comparative studies between commercial platforms remain scarce.^35^^,^^36^

Comparisons of bottle types within the same commercial platform provide practical insights for clinical use. Fungus-specific bottles such as BACTEC Mycosis IC/F demonstrate greater sensitivity for *C. albicans* and *C. glabrata*, with consistently shorter times to positivity. Nonetheless, Mycosis IC/F bottles are not reliable for monitoring clearance of fungemia. It’s more reliable recovery for the initial diagnosis of candidemia, especially in high-risk patients not yet exposed to antifungal treatment.^35^^,^^37^

In summary, the panel recommends the use of automated blood culture systems for the diagnosis of candidemia (AII evidence), prioritizing conventional bottles capable of detecting both bacteria and fungi, given the higher frequency of bacterial bloodstream infections and the reliable growth of *Candida* species in standard media (BII evidence). A fungus-specific bottle should be added to the initial diagnostic blood culture set in high-risk patients, particularly those with suspected intra-abdominal candidiasis, recent liver transplantation, hematologic malignancies receiving chemotherapy with prolonged neutropenia, or chronic hemodialysis, respecting local epidemiology and the availability of financial resources. Patients selected to receive empirical antifungal therapy may also benefit from fungal-specific blood culture bottles (CIII evidence).^35^, ^36^, ^37^, ^38^, ^39^

### What is the recommended blood volume and number of samples to be collected in blood cultures to increase the sensitivity of candidemia diagnosis in adults?

Several pre-analytical and analytical variables affect the detection of *Candida* species, most notably the blood volume collected, the number of culture sets, and the type of bottles employed. The overall sensitivity of blood cultures for candidemia is low, averaging 40% and ranging from 21%‒71% depending on the infectious inoculum, infection source, host factors, bacterial coinfection, and prior antifungal exposure. Expert consensus advises collection of two culture sets (four bottles in total), each filled with 10 mL of blood, obtained sequentially from distinct venipuncture sites to optimize recovery and reduce false-negative results. Blood culture diagnostic performance improves as both the volume of blood obtained and the number of incubated bottles increase (AII evidence).^40^ Adherence to institutional protocols for sample collection and processing remains essential to ensure diagnostic accuracy (AII evidence).^40^, ^41^, ^42^, ^43^, ^44^

### What is the recommendation regarding follow-up blood cultures after the diagnosis of candidemia?

Repeating blood cultures after the diagnosis of candidemia is essential for two main reasons. First, documenting microbiological clearance is critical, as persistent fungemia may signal treatment failure, antifungal resistance, or uncontrolled foci such as endocarditis, abscesses, or contaminated devices. Despite limited sensitivity, blood cultures remain the gold standard for confirming clearance, and persistent positivity has been linked to increased mortality and metastatic complications. Second, repeat cultures guide treatment duration, with international consensus recommending at least 14-days of antifungal therapy after the first negative blood culture and clinical resolution. This conservative approach generally extends therapy by one to two days compared with counting from the last positive culture, but it remains the standard endorsed by current guidelines. However, obtaining daily blood cultures may be challenging in routine clinical practice; therefore, the panel recommends follow-up blood cultures on the third and fifth days after initiation of therapy as a reasonable and accepted strategy for the purposes described above (BIII evidence). Additional blood cultures are justified only if these follow-up cultures remain positive (AIII evidence).^40^^,^^43^^,^^44^

### What is the recommendation for the use of non-culture-based diagnostic methods, such as antigen detection tests, 1,3-Beta-D-Glucan detection, or Polymerase Chain Reaction (PCR) tests, in the diagnosis of candidemia?

Non-culture-based diagnostic tests, including 1,3-β-D-Glucan (BDG) and PCR-based assays, should be regarded as adjuncts rather than replacements for blood cultures. While they can provide earlier results, improve negative predictive value, and assist in excluding IC in patients with low-to-moderate pretest probability, they cannot establish a definitive diagnosis or provide species identification. Their positive predictive value is limited in low-prevalence settings, requiring cautious interpretation. Negative results, however, may support discontinuation of empiric antifungal therapy. For these reasons, current recommendations advise targeted rather than routine use, focusing on patients with moderate-to-high suspicion of invasive candidiasis.^40^^,^^45^, ^46^, ^47^, ^48^, ^49^ These methods can provide earlier results and improve the negative predictive value (CII evidence). However, it should be noted that these diagnostic methods are mostly unavailable in medical centers in Brazil.^29^

Molecular assays can detect *Candida* DNA in whole blood earlier than blood cultures, with an average lead time of approximately two days. Most PCR-based methods for candidemia are in-house assays, with a lack of clinical standardization and variable performance across laboratories, which limits their applicability. When used, *Candida* PCR should only serve as a complementary tool alongside conventional diagnostic. The panel does not recommend the routine use of PCR in whole blood for the diagnosis of candidemia (DIII evidence).^50^, ^51^, ^52^, ^53^ Currently, multiplex PCR platforms targeting the main pathogens responsible for sepsis, including bacteria and a limited number of *Candida* species, are commercially available. However, these systems still require a positive blood culture bottle to perform species identification, which substantially limits their utility for the diagnosis of candidemia (CIII evidence).^53^

BDG is a pan-fungal biomarker with reported sensitivity for candidemia of 78%‒88% but lower specificity due to false positives related to other fungal infections, heavy *Candida* colonization, exposure to blood products, certain antibiotics, and hemodialysis. Positivity often precedes blood culture detection, and rising levels may indicate relapse or poor outcomes. Its main value lies in a high negative predictive performance, particularly in low-to-moderate prevalence settings. BDG should never be used as a stand-alone diagnostic tool, and results must be interpreted considering the clinical context and possible confounders (EIII evidence).^48^, ^49^, ^50^, ^51^, ^52^, ^53^

In summary, despite decades of investigation into novel diagnostic approaches, the diagnosis of candidemia in Brazil remains essentially restricted to fungal detection by blood culture.

### How to identify *Candida* at the species level?

Accurate species-level identification of *Candida* remains challenging, particularly in low- and middle-income settings where most laboratories lack access to automation or mass spectrometry. This limitation hampers timely differentiation of clinically relevant species and may delay the initiation of targeted antifungal therapy. In this context, three levels of diagnostic complexity can be envisioned, each with validated utility in the medical literature. These range from basic presumptive methods to automated biochemical systems and, ideally, mass spectrometry, reflecting the diversity of resources and capacities across institutions. At the most basic level, institutions without automation, MALDI-TOF, or genomic platforms may rely on presumptive culture media such as Chromagar *Candida* Plus® (complemented by microscopy findings) to provide preliminary genus and species-level screening (AIII evidence). In these settings, referral of invasive isolates to reference laboratories should be encouraged.^54^, ^55^, ^56^

A second level includes hospitals with automated biochemical systems such as Vitek®2, Phoenix™, or Microscan®, which are faster, less labor-intensive, and broadly available; multiple evaluations over the past decade support their validity and reliability for routine *Candida* identification (AII evidence). Reported performance includes high correct identification rates for common yeasts and turnaround often within 18h (from colony availability), supporting their clinical utility where MALDI-TOF is not accessible. Nonetheless, accuracy for emerging or uncommon species depends on regularly updated databases.^57^^,^^58^

The highest level of complexity involves laboratories equipped with MALDI-TOF mass spectrometry, the current method of choice, which enables rapid and accurate identification within minutes after colony growth and can distinguish closely related species. This technology is particularly valuable for critically ill patients, for detecting accurately a large number of fungal species, including emerging and resistant species such as *C. auris*, in a short period of time. Genus and species-level identification by proteomics (MALDI-TOF), supported by regularly updated libraries, is recommended over automated or semi-automated biochemical methods, whenever locally available (AI evidence).^59^^,^^60^

### What is the recommendation for performing antifungal susceptibility testing in *Candida* species, and how do the different available methods perform?

The growing use of systemic antifungals has not been matched by improved outcomes, and the emergence of resistant isolates underscores the importance of Antifungal Susceptibility Testing (AFST) to guide therapy and stewardship. Broth microdilution, preferably following EUCAST methodology, is the recommended standard. In Brazil, EUCAST guidelines are applied through BrCAST, whose use has been mandatory in all laboratories since 2018. BrCAST provides freely accessible documents with updated breakpoints, ECOFFs, and guidance for interpreting Minimum Inhibitory Concentrations (MICs), including recommendations for rare yeasts not covered in standard tables available at https://brcast.org.br. 61, 62, 63

Comparing and validating commercial methods against the broth microdilution reference standard remains challenging due to methodological differences and limited standardization. Nevertheless, automated systems such as Vitek2, colorimetric assays like Sensititre™ YeastOne, and gradient diffusion strips for MIC determination have demonstrated high essential agreement across widely used antifungals, including amphotericin B, fluconazole, voriconazole, micafungin, and anidulafungin, reinforcing their reliability for use in clinical practice.^63^, ^64^, ^65^

Taking in consideration the epidemiology of candidemia in Brazil, screening for azole resistance with fluconazole is generally sufficient. Surveillance data indicate that echinocandin resistance remains rare, including in *C. glabrata*.^66^ When performed, screening of echinocandin resistance can be done with micafungin or anidulafungin. It should also be emphasized that no BrCAST clinical breakpoints exist for caspofungin and that the disk diffusion is not recommended for antifungal susceptibility testing.^67^, ^68^, ^69^

The greatest clinical value of AFST lies in invasive infections, particularly when isolates are recovered from blood, sterile fluids, or deep-seated sites. Beyond these contexts, testing is especially useful in patients with prior antifungal exposure, clinical failure or breakthrough disease, and in the presence of rare, and emerging yeasts potentially associated to multi-resistance as *C. auris* (AII evidence).

It is also warranted when surveillance in specific centers indicates unusual resistance patterns in typically susceptible species, as observed with fluconazole-resistant *C. parapsilosis* or *C. tropicalis* (BII evidence).^63^^,^^70^

**When is a fundoscopic (eye) examination indicated in patients with candidemia?**

The choice and the duration of antifungal therapy can be influenced by ocular candidiasis with vitreous involvement. However, due to the low incidence of *Candida* endophthalmitis, routine fundoscopy is no longer universally recommended for all patients with candidemia.^40^^,^^71^, ^72^, ^73^, ^74^ Instead, it is advised for:

• Patients with ocular symptoms, especially visual complaints (AII evidence);
• High-risk populations, such as those with persistent candidemia, under chronic hemodialysis (more than 3-months),^75^ immunosuppressed individuals (AII evidence);
• Patients unable to verbalize symptoms (e.g., ICU patients on mechanical ventilation) (AII evidence).
• A new fundoscopic examination of the eye may be considered at any time in symptomatic patients, those not responding to treatment as well as hematologic patients after recovering from neutropenia (AIII evidence).^40^, ^76^

### What is the indication for performing an echocardiogram in cases of candidemia?

Candidemia-associated endocarditis presents high morbidity and mortality, and timely identification can impact decisions regarding surgical intervention and duration of antifungal therapy.^77^

The indication for performing echocardiography in all patients with candidemia remains controversial. Recent evidence supports a risk-based approach that avoids unnecessary testing in low-risk individuals. Current invasive candidiasis guidelines recommend echocardiography, preferably transesophageal, in patients with identifiable risk factors for *Candida* endocarditis, as endocarditis is documented in less than 4 percent of patients with candidemia in most large series.^40^^,^^78^^,^^79^

A Brazilian study reported that echocardiography was performed in 63.7% of candidemia cases, identifying infective endocarditis in 4.9% of patients.^80^ The authors advocate for targeted echocardiographic screening, focusing on high-risk patients or those with persistent positive blood cultures, rather than universal application.

Therefore, routine echocardiography is no longer universally recommended for all patients with candidemia, but it is strongly recommended for patients with ate least one of the following risk factors for endocarditis:^81^, ^82^, ^83^, ^84^, ^85^

• Presence of new cardiac murmurs or peripheral embolic phenomena (e.g., skin lesions) (AII evidence);
• Pre-existing valvar disease, prosthetic valves, or presence of cardiac implantable electronic devices (AII evidence);
• Patients under chronic hemodialysis (more than 3-months) (AII evidence);^75^
• Illicit use of intravenous injection drugs (AII evidence);
• Persistent candidemia despite adequate antifungal therapy (AII evidence).

## Treatment

### When should empirical antifungal therapy for candidemia be initiated in critically ill patients, and what is the role of *Candida* scores in guiding empirical therapy?

Randomized controlled trials have not demonstrated a clear survival benefit of patients submitted to empirical antifungal therapy, possibly due to the difficulty in predicting candidemia and, consequently, the low number of IC cases enrolled in these studies.^86^^,^^87^ Although risk factors for candidemia are widely prevalent in the hospital setting, existing candidemia prediction rules consistently exhibit high sensitivity but low specificity. However, observational studies consistently show that delays in initiating antifungal therapy in culture-proven candidemia are associated with increased mortality.^88^^,^^89^ This creates a dilemma: starting empirical therapy too broadly increases unnecessary drug exposure, resistance selection, and costs, while delaying treatment in true cases of candidemia worsens outcomes. In this scenario, once empirical antifungal therapy is empirically initiated, the clinical staff should re-access the patient after 3‒5 days for checking culture and biomarker results to decide if ATF should be maintained or removed faced new clinical and laboratory findings obtained at that time.^90^

Due to all controversies mentioned above, the panel understands that empirical antifungal therapy should be mostly considered for patients with septic shock or for those with clear clinical deterioration in the presence of established risk factors for candidemia, including prolonged intensive care unit stay, exposition to broad spectrum antibiotics, intravascular catheterization, especially when *Candida* colonization is documented in multiple sites (CIII evidence).^40^^,^^44^ For other individuals, empirical antifungal therapy should be avoided, and close monitoring should be prioritized.

### When and how should empirical antifungal therapy for candidemia be discontinued?

Empirical antifungal therapy in critically ill patients must be discontinued when there is sufficient evidence to rule out IC and when clinical parameters indicate that ongoing treatment is unnecessary. The inappropriate continuation of antifungals contributes to drug resistance, toxicity, and increased healthcare costs. Given the absence of high-quality randomized controlled trials demonstrating a survival benefit of empirical therapy, its duration should be minimized whenever possible. The challenge is to balance early discontinuation in patients unlikely to have IC while ensuring appropriate treatment for those truly infected.^40^^,^^44^

In settings where BDG- or PCR-based methods are available, sequential negative BDG tests or negative PCR results could guide stopping antifungal therapy. Studies have demonstrated that discontinuing antifungal therapy after two or three consecutive negative biomarkers is safe, with no increased risk of mortality or relapse of candidemia.^91^, ^92^, ^93^, ^94^, ^95^, ^96^

In the absence of fungal biomarkers, discontinuation must rely on clinical and microbiological reassessment. After 3‒5 days of empirical therapy, the patient should be reassessed and checked for the presence of: (i) A new source of infection that was not identified at the beginning; (ii) Isolation of MDR bacteria not treated appropriated initially; (iii) The clinical status of the patient and the continuous exposition to risk factors for IC; (iv) New blood cultures for *Candida*, positive fungal biomarker or other evidence of deep-seated *Candida* infection; and (v) Multicolonization by *Candida* (AIII evidence). Pending on the clinical and laboratory information generated along this reassessment, clinician should consider in individually bases if empirical therapy should be removed or continued (BIII evidence).^91^^,^^97^, ^98^, ^99^, ^100^

### What are the antifungal agents of choice for initial empirical, and microbiologically documented candidemia treatment?

Echinocandins (anidulafungin, caspofungin, micafungin, and rezafungin) are the preferred first-line antifungal agents for the treatment of laboratory-documented candidemia, given their broad-spectrum activity, fungicidal effect, activity against biofilm, and favorable safety profile (AI evidence). Clinical trials and real-world data consistently demonstrate their superior efficacy compared to fluconazole, especially in critically ill or unstable patients, including those with prior azole exposure.^101^^,^^102^ All echinocandins of first generation are apparently similar in terms of efficacy, as head-to-head trials comparing micafungin and caspofungin found no significant difference in treatment success, time to blood culture clearance, or mortality.^103^ However, pharmacokinetic and pharmacodynamic differences suggest micafungin or anidulafungin may be preferred in patients with moderate hepatic impairment, as caspofungin requires dose adjustment in hepatic dysfunction (AII evidence). Moreover, anidulafungin is unique among first-generation echinocandins for its lack of hepatic metabolism, making it preferable in patients with significant hepatic dysfunction. Micafungin, among the echinocandins, has the advantage of not requiring a loading dose, unlike anidulafungin or caspofungin. Micafungin demonstrates greater stability after reconstitution and does not require refrigeration, allowing storage at room temperature for up to 24 hours.^104^

Rezafungin, a long-acting echinocandin with a prolonged half-life, offers the advantage of once-weekly dosing and has demonstrated non-inferiority to caspofungin in clinical trials.^105^, ^106^, ^107^, ^108^ This makes it an attractive alternative in scenarios where outpatient treatment or reduced healthcare burden is desirable. Clinical trials have demonstrated non-inferiority of rezafungin compared to caspofungin in terms of all-cause mortality at day-30.^109^^,^^110^ Interestingly, rezafungin demonstrated higher rates of early mycological eradication compared with caspofungin in a pooled analysis of trials phase II and III, which may be related to the higher plasma exposures achieved with this agent.^110^ Additionally, rezafungin exhibits enhanced stability and reduced hepatic metabolism, potentially reducing drug-drug interactions and making it a compelling option for critically ill patients with hepatic dysfunction or on complex medication regimens.^111^

Empirical therapy for suspected candidemia should follow the same principles as targeted therapy for blood culture-proven infection and should therefore be initiated with an echinocandin. However, due to the long half-life of rezafungin, the panel has concerns to use this agent as first-line empirical antifungal therapy, as its prolonged pharmacokinetics preclude early clinical reassessment within 3‒5 days and timely discontinuation once antifungal therapy is no longer indicated (CIII evidence).

### When and how should step-down therapy with azoles be performed in patients with candidemia?

Sequential therapy, also referred to as step-down therapy or de-escalation therapy, in the management of candidemia involves transitioning from an initial broad-spectrum intravenous antifungal agent, typically an echinocandin, to a narrower-spectrum oral antifungal, usually fluconazole. This approach is employed once the patient has shown clinical improvement, and the *Candida* species is identified as susceptible to the oral agent. The primary goals are to reduce the duration of intravenous therapy, minimize hospital stays, decrease treatment costs, and lower the risk of complications associated with intravenous catheters, all while maintaining therapeutic efficacy.^40^^,^^44^

Observational studies suggest that de-escalation from echinocandin therapy to oral fluconazole is safe.^112^, ^113^, ^114^ This transition generally occurs after 5‒7 days of intravenous echinocandin therapy, though it may be adjusted based on patient factors. The rationale for sequential therapy with azoles is supported by evidence showing that early effective treatment improves outcomes in candidemia, while de-escalation reduces unnecessary antifungal exposure and healthcare costs.^115^, ^116^, ^117^ Of note, fluconazole should not be used as first line therapy in critically ill patients or when *C. glabrata* or *C. krusei* is suspected, as these species exhibit decreased susceptibility or resistance (AIII evidence). Liposomal amphotericin B (L-AmB) (3‒5 mg/kg/day) remains a second-line option for resistant or refractory infections, particularly in azole- or echinocandin-resistant *C. glabrata* or *C. auris* cases (AII evidence).^40^

Sequential therapy with fluconazole (400‒800 mg daily) should be initiated when the following criteria are met (BII evidence):

• Clinical and laboratorial improvement: The patient exhibits signs of clinical stability, such as resolution of fever, stabilization of vital signs (e.g., blood pressure, heart rate), and improvement in laboratory parameters (e.g., white blood cell count, C-reactive protein, negative blood cultures);
• AFST should be performed whenever possible for all bloodstream *Candida* isolates, using any validated commercial method. In settings where universal testing is not feasible, susceptibility testing is strongly recommended for non-*C. albicans* species, particularly those with variable or reduced susceptibility. For species with predictable susceptibility profiles, such as *C. albicans*, step-down therapy may be considered once species identification is established;^118^
• Absence of metastatic complications: There should be no evidence of complications such as endocarditis, osteomyelitis, or other deep-seated infections that would require prolonged intravenous therapy. Such complications necessitate extended intravenous treatment to ensure adequate penetration and control of infection;^119^
• Ability to tolerate oral medications: The patient must be capable of taking oral medications and have adequate GI absorption. This ensures that the oral antifungal achieves therapeutic levels.^116^

### What are the criteria for persistent candidemia? When should a deep-seated infection be investigated?

Persistent candidemia is defined as the continued presence of *Candida* spp. in blood cultures despite appropriate antifungal therapy, typically beyond 5-days of treatment.^120^^,^^121^ It represents a critical clinical scenario that may impact mortality and frequently signals an underlying uncontrolled source of infection.^118^ The persistence of candidemia can be attributed to several factors, including delayed or inadequate source control, high fungal burden, the presence of biofilm-associated infections (such as intravascular catheters or prosthetic devices), inadequate immune response, or antifungal resistance, particularly in non-*C. albicans Candida species* such as *C. glabrata* and *C. auris*, which frequently exhibit multidrug resistance.^118^, ^122^, ^123^ Identifying patients at risk for persistent candidemia is essential, as these individuals may require more aggressive management strategies, including prolonged therapy, surgical interventions as well as intensive work-up for checking secondary localization of *Candida* infection.

Investigation of deep-seated infection should be initiated in all cases of persistent candidemia (AII evidence). Recommended diagnostic approaches include transesophageal echocardiography to evaluate for *Candida* endocarditis, ophthalmologic examination to assess for endophthalmitis, and imaging studies to identify hepatosplenic candidiasis, osteomyelitis, vascular thrombosis or other forms of disseminated disease.^40^^,^^72^^,^^124^, ^125^, ^126^ Indeed, secondary localization was found in 21.8% of 376 ICU patients with candidemia screened for secondary localization of fungal lesions in 16 French ICUs. In this multicenter study, deep seated candidiasis was independently associated with disease severity, prolonged fungemia, longer antifungal treatment, and higher rates of therapy escalation.^127^ Intravascular catheters should be promptly removed if suspected as the source of infection, and other potential reservoirs, such as infected prosthetic materials, should be considered. Failure to control the infection through targeted interventions can significantly worsen patient outcomes, necessitating a comprehensive approach to antifungal management and source control.^40^, ^44^

### What is the recommended duration of antifungal therapy for candidemia?

The duration of antifungal therapy for candidemia varies depending on the clinical scenario, such as the presence of complications, persistence of infection, or specific patient conditions. For candidemia without metastatic complications (e.g., endocarditis, endophthalmitis, osteomyelitis), persistent positive blood cultures, or complicating conditions, the duration should be 14-days from the first negative blood culture, even though this recommendation comes mainly from expert opinion, based on limited data (AIII evidence).^128^ In candidemia in neutropenic patients, duration should be at least 14-days after the first negative blood culture and until neutrophil recovery (AIII evidence). Neutropenic patients are at higher risk for dissemination, and immune recovery is essential for infection control. Clearance should be ensured (and used for a reference for duration) through follow-up cultures.^129^

In complicated candidemia with metastatic foci (candidemia with dissemination to organs, such as endocarditis, endophthalmitis, or osteomyelitis), extended therapy should be tailored to the specific complication. For endocarditis: at least 6-weeks, often combined with surgical intervention (BII evidence).^119^ For endophthalmitis (vitreous involvement): 4‒6 weeks, potentially with intravitreal antifungals (BIII evidence).^130^ For osteomyelitis, 6-months, typically requiring surgical debridement alongside antifungal therapy (BIII evidence).^131^ In these situations, prolonged therapy is necessary to eradicate infection from tissues with poor antifungal penetration. Inadequate treatment duration in complicated cases increases the risk of delayed complications.^119^^,^^131^

Source control remains critical. Delays in effective therapy and source control are associated with increased mortality.^40^, ^129^ Currently, there are several investigators discussing if a shorter period of treatment would be possible in a setting of non-complicated patients.^132^^,^^133^

### What are the criteria for therapeutic failure and the indication for switching to salvage therapy in patients with candidemia?

Given the high mortality associated with candidemia, timely recognition of therapeutic failure and initiation of rescue therapy is essential for improving clinical outcomes.^134^

Key indicators of failure include persistent positive blood cultures beyond five days of treatment, lack of clinical improvement, the development of new metastatic foci of infection, or progression to sepsis or septic shock.^121^^,^^135^ Factors contributing to treatment failure include severity of illness, presence of multiple comorbidities, delayed initiation of antifungal therapy, inappropriate initial drug or dosage regimen selection, high fungal burden, biofilm-associated infections (such as intravascular catheters or prosthetic devices), inadequate source control, and antifungal resistance.^40^^,^^136^^,^^137^ In a recent multicenter retrospective study, failure with echinocandins were associated with obesity, septic shock, and increased MICs to echinocandins.^137^ *C. glabrata, C. auris*, and *C. parapsilosis* have shown increasing resistance to fluconazole and, in some cases, to echinocandins, significantly impacting treatment outcomes. IAC without adequate source control, infective endocarditis, and deep thrombophlebitis may represent additional causes of persistent infection and should prompt thorough diagnostic investigation.^138^, ^139^, ^140^

Rescue therapy should be promptly initiated in patients meeting criteria for therapeutic failure. This includes escalation to an alternative antifungal of a different class, such as switching from fluconazole to an echinocandin or from an echinocandin to L-AmB (AII evidence), particularly in cases of suspected or confirmed resistance.^138^ Additionally, source control is paramount ‒ this includes removing CVCs,^135^^,^^141^ draining abscesses, and surgically addressing infective endocarditis or osteomyelitis if present.^135^^,^^138^ Persistent candidemia should prompt a reassessment of antifungal susceptibility, as resistance patterns may emerge during treatment, necessitating tailored therapeutic adjustments.^136^^,^^142^^,^^143^

Some patients represent difficult-to-treat populations (Table 4), such as those with obesity, critical illness, exposure to Extracorporeal Membrane Oxygenation (ECMO), or marked third-space fluid shifts, and may require dose escalation and/or therapeutic drug monitoring to ensure adequate antifungal exposure (BII evidence).^144^ Due to concerns with suboptimal plasma levels in critically ill patients receiving first generation echinocandins (anidulafungin, micafungina, and caspofungin), as well as the promising results of rezafungin reducing the time to clear blood cultures when compared to caspofungin, some authors have suggested that higher doses of echinocandins may have a play in salvage therapy (CIII evidence).^79^^,^^145^, ^146^, ^147^, ^148^

**Table 4:** Management of invasive candidiasis in special populations.

Special population	Key pharmacokinetic (PK) considerations	Recommended antifungal adjustments and strategies
Obesity	Increased volume of distribution and altered clearance for lipophilic drugs like fluconazole. Increased clearance for echinocandins correlated with body size.	**Echinocandins** are the drugs of choice. Consider dose optimization for **micafungin** and **caspofungin. Fluconazole** requires accurate weight-adjusted dosing.
Advanced age	High mortality risk associated with multiple comorbidities. Potential for reduced drug clearance.	Dose adjustments are often necessary for renally cleared drugs, particularly **fluconazole (Creatinine Clearence <50mg/dL)**.
Renal insufficiency and dialysis	Reduced clearance of drugs eliminated via the kidneys. Low removal of echinocandins and L-AmB by dialysis circuits.	Adjust **fluconazole** dosage based on renal function/dialysis. **No adjustment** is required for echinocandins or **Liposomal Amphotericin B (L-AmB)**.
Hepatic failure	Risk of drug accumulation for hepatically metabolized agents.	**Caspofungin:** Reduce to 50 mg daily in moderate impairment (Child-Pugh B). **Fluconazole:** Generally safe in mild/moderate cases; use with caution in severe (Child-Pugh C). **No adjustment** needed for micafungin, anidulafungin, rezafungin, or L-AmB.
ECMO support	Drugs (especially lipophilic or highly protein-bound like azoles) may be sequestered by the ECMO circuit, leading to sub-therapeutic levels.	**Echinocandins** and **L-AmB** are preferred due to better PK stability compared to triazoles. Treatment must be **individualized** based on host physiology and drug properties.

ECMO, Extracorporeal Membrane Oxygenation; L-AmB, Liposomal amphotericin B.

### What is the role of combination antifungal therapy in the treatment of candidemia?

Combination antifungal therapy involves using two or more antifungal agents simultaneously to treat IC, a bloodstream infection caused by *Candida* species. While monotherapy is typically the standard approach, combination therapy has been studied for the potential to improve outcomes in specific situations. It is not a valid approach for most patients due to insufficient evidence of broad benefits and potential risks. Combination antifungal therapy could have a targeted role in treating candidemia, particularly in resistant, difficult-to-treat, species-specific, or refractory cases.^149^, ^150^, ^151^

A comparison of high-dose fluconazole plus placebo to fluconazole plus amphotericin B in non-neutropenic patients with candidemia found no significant outcome difference, indicating limited benefit in uncomplicated cases.^152^ Ostrosky-Zeichner et al. (2005) found that micafungin, alone or combined with other agents, was effective in some refractory candidemia cases, though broader conclusions were limited.^153^ Further research is necessary to explore the potential of combination therapy in refractory infections.^154^

The panel recommends against using combination therapy in patients with candidemia, due to lack of proven benefit and potential drawbacks, including costs and toxicity (DII evidence). Otherwise, combination therapy (e.g., echinocandin or 5-flucytosine plus amphotericin-B) may be warranted for severe cases, such as infective endocarditis or Central Nervous System (CNS) involvement (BIII evidence).^40^, ^149^, ^150^ In refractory cases, difficult-treat and significant resistance situations, more data is needed to ensure the recommendation.

### How should candidemia be managed in special populations: obesity, advanced age, renal insufficiency, hepatic insufficiency, dialysis, and ECMO?

Table 4 summarizes our main recommendations for the treatment of candidemia in special populations, such as patients with obesity, advanced age, renal or hepatic dysfunction, dialysis dependence, or those supported by ECMO. These conditions can substantially alter drug absorption, distribution, metabolism, and clearance. For example, patients with obesity may exhibit increased volume of distribution and altered clearance for lipophilic antifungals such as fluconazole, necessitating weight-based dosing strategies to achieve therapeutic exposure.^155^, ^156^, ^157^ A correlation also exists between increased body size and increased clearance for the echinocandins, so higher doses may be required for the heavier patients.^158^^,^^159^ In elderly patients, candidemia has been associated with a high mortality rate, particularly due to the presence of comorbidities.^160^ Dose adjustment is often necessary in patients with renal impairment or on dialysis when drugs are renally cleared, particularly for fluconazole.^159^ In contrast, echinocandins and L-AmB do not require adjustment in renal dysfunction. Patients with hepatic insufficiency may also accumulate hepatically metabolized drugs.^161^ Fluconazole is primarily eliminated renally, with partial hepatic metabolism, and generally does not require dose adjustment in mild to moderate hepatic dysfunction (Child-Pugh A or B); however, caution is advised in severe hepatic impairment (Child-Pugh C) due to potential hepatotoxicity, and liver function should be closely monitored. Among the echinocandins, caspofungin requires dose reduction to 50 mg daily in moderate hepatic impairment, while micafungin and anidulafungin do not require adjustment even in severe cases.^144^ Rezafungin, a long-acting echinocandin with primarily non-hepatic clearance, does not require dose modification in hepatic impairment based on current pharmacokinetic data. L-AmB, which undergoes minimal hepatic metabolism and is not significantly excreted via the biliary route, also does not require dose adjustment in any degree of hepatic dysfunction. Moreover, in patients undergoing ECMO, antifungal agents, especially lipophilic or highly protein-bound drugs such as azoles, may be sequestered by the circuit,^162^^,^^163^ potentially resulting in sub-therapeutic plasma levels and treatment failure.^164^ These complex scenarios demand not only attention to dosing but also to drug selection, particularly to minimize toxicity in organ dysfunction settings.

Echinocandins are the preferred first-line agents across most of these special populations due to their favorable pharmacokinetics, minimal renal or hepatic clearance, low toxicity profiles, and negligible removal by dialysis or ECMO circuit.^144^ No dose adjustments are necessary for renal dysfunction, including patients on hemodialysis or continuous renal replacement therapy, or in those with mild to moderate hepatic impairment. However, in severe hepatic dysfunction, caspofungin may require cautious use due to mild hepatic metabolism. L-AmB offers a broad spectrum and remains an effective alternative in cases of suspected or proven resistance but should be used cautiously in renal impairment given its nephrotoxic potential. In patients with obesity, echinocandins remain the drug of choice, though some pharmacokinetic models suggest considering dose optimization, particularly for micafungin and caspofungin.^165^ As for fluconazole, studies show that inadequate dosing is common, particularly in patients with elevated creatinine clearance or higher body weight, underscoring the need for accurate, weight- and renal function-adjusted dosing when this agent is used.^155^ Notably, emerging agents such as rezafungin, administered once weekly with a long half-life and potent activity against resistant *Candida* spp. have shown promise for use in critically ill or complex populations. Data generated so far suggest thaty rezafungin does not need dose adjustment in obese, elderly or renally impaired patients, but further validation in special populations remains warranted. In ECMO-supported patients, echinocandins and L-AmB have demonstrated better pharmacokinetic stability compared to triazoles, which may be adsorbed by the ECMO membrane.^162^^,^^163^ Therefore, treatment of candidemia in these patients must be individualized (Table 4), with antifungal selection and dosing guided by host physiology, drug properties, and pathogen susceptibility (AIII evidence).

### When is recommended the removal of a central venous catheter in patients with candidemia?

The removal of a CVC is recommended promptly upon diagnosis of candidemia, particularly when the catheter is suspected to be the source of the infection. This recommendation stems from the understanding that CVCs are a frequent nidus for *Candida* infections due to biofilm formation, where fungi adhere to the catheter surface, making eradication more difficult without removal. Prompt removal, typically within 24 to 48 hours of diagnosis, is supported by multiple observational studies that associate delayed removal with persistent infection and increased mortality.^47^^,^^166^, ^167^, ^168^, ^169^

Several studies highlight the benefits of early CVC removal.^170^, ^171^, ^172^, ^173^ Garnacho-Montero et al. found that catheter removal, combined with adequate antifungal therapy, significantly reduced hospital mortality in patients with *Candida* bloodstream infections.^170^ Moreover, retaining the CVC can lead to persistent candidemia, as seen in a retrospective cohort study from Korea, often due to the catheter acting as a continuous source of fungal dissemination.^174^

While prompt removal is the default recommendation, there are scenarios where retaining the CVC may be considered, including non-catheter-related candidemia and situations where maintenance of the CVC is essential and/or removal is risk or not feasible.^175^ If the infection is clearly from another source (e.g., GI translocation), removal may not be necessary.^176^ Some conflicting evidence, indeed, found that early removal did not improve outcomes^177^ raising questions about universal removal, or suggest a more individualized approach.^178^ A Cochrane review demonstrated the absence of randomized controlled trials directly comparing removal versus retention, indicating that the evidence base relies heavily on observational data, which, however, largely favors removal.^141^

In clinical practice, prompt removal typically means within 24‒48 hours of confirming candidemia via blood cultures, balancing the urgency of intervention with the need to stabilize the patient and arrange alternative vascular access if required (AI evidence). Studies referring to early removal^173^^,^^179^^,^^180^ often define this window, though exact timing may vary based on clinical context.

### What is the importance of the infectious disease specialist in the clinical management of candidemia?

The management of candidemia is complex and requires adherence to multifaceted diagnostic and therapeutic interventions that directly influence clinical outcomes. Infectious diseases specialists play a crucial role in guiding early and appropriate antifungal therapy, identifying and controlling infection sources, recommending adjunctive diagnostics (e.g., echocardiography, fundoscopy), and ensuring adherence to evidence-based guidelines.^181^, ^182^, ^183^, ^184^ Several studies have highlighted the association between early infectious diseases consultation and improved candidemia outcomes, including reductions in mortality, enhanced guideline adherence, and increased likelihood of timely source control interventions such as central line removal.^181^^,^^182^^,^^184^ Furthermore, infectious diseases consultation has been shown to reduce inappropriate antifungal use, decrease unnecessary therapy durations, and promote antifungal stewardship, contributing to both clinical and economic benefits.^185^^,^^186^

Data from large cohort studies demonstrate that infectious diseases consultation significantly improves survival in patients with candidemia. For instance, in a retrospective cohort of over 1,600 patients with candidemia, those who received infectious diseases consultation had a 19% lower risk of 90-day mortality (hazard ratio 0.81; 95% Confidence Interval 0.73–0.91) and were more likely to receive timely antifungal treatment, echocardiography, ophthalmologic evaluation, and catheter removal.^187^ Similar findings were reported in a Japanese study, where infectious diseases consultation was associated with a 46% reduction in 30-day mortality (adjusted hazard ratio 0.54; 95% Confidence Interval 0.32–0.90).^183^ A study conducted in Brazil revealed that infectious disease specialists demonstrated superior knowledge and practices in the management of candidemia compared to ICU physicians, including a better understanding of disease epidemiology and a more appropriate preference for echinocandins as first-line therapy in unstable patients.^188^ Among cancer patients with *C. glabrata* fungemia, infectious diseases involvement resulted in earlier initiation of appropriate antifungals and better outcomes, especially in non-catheter-associated cases.^189^ Collectively, these findings reinforce the importance of routine and early involvement of infectious diseases specialists in the management of candidemia, not only to reduce mortality but also to ensure optimal and cost-effective care. Given the high morbidity, mortality, and resource utilization associated with candidemia, systematic infectious diseases consultation should be considered a standard of care (AII evidence).

It should be reinforced that a detailed dermatological examination should also be performed in these individuals. Although cutaneous involvement in candidemia is uncommon, skin lesions may provide an important diagnostic clue.

## Invasive candidiasis in other sites

### What is the classification of intra-abdominal candidiasis?

Intra-abdominal candidiasis (IAC) comprises a heterogeneous group of infections, with varying extent and severity, including secondary or tertiary peritonitis, intra-abdominal abscesses, and necrotic or purulent complications related to GI perforation or anastomotic leakage.^40^^,^^190^^,^^191^ IAC typically occurs in surgical patients following abdominal procedures, particularly in cases of necrotizing pancreatitis, delayed or incomplete source control, or recurrent GI leakage. Although candidemia is a defining feature of IC, it is detected in fewer than 1 in 5 cases of IAC, highlighting the need for diagnostic criteria specifically tailored to deep-seated infections.^192^, ^193^, ^194^

Currently, IAC is defined as a form of deep-seated candidiasis and classified into proven and probable IAC.^190^ Proven IAC is defined by histological or microbiological identification of *Candida* spp. in sterile intra-abdominal samples (e.g., peritoneal fluid or abscess), obtained via surgery or image-guided aspiration, in the absence of recent GI perforation. Probable IAC requires one clinical and one mycological criterion. Acceptable mycological findings include the isolation of *Candida* spp. from intra-abdominal specimens obtained >24h after a perforation or from recurrent peritonitis. Clinical criteria include radiologic evidence compatible with IAC not otherwise explained.^40^^,^^190^^,^^193^ In the postoperative setting, IAC most often results from secondary or tertiary peritonitis, particularly after upper GI perforation. When *Candida* is detected in peritoneal fluid of patients with postoperative sepsis and persistent fever, a fungal etiology should be strongly considered. Antifungal susceptibility testing of multiple intra-abdominal isolates is encouraged.^195^

Continuous ambulatory peritoneal dialysis-associated *Candida* peritonitis is a serious complication in patients undergoing peritoneal dialysis, representing one of the most significant infectious challenges in this population. Key risk factors include diabetes, previous episodes of bacterial peritonitis, and recent antibiotic exposure.^40^^,^^190^^,^^195^, ^196^, ^197^ Timely diagnostic suspicion, catheter removal, and antifungal therapy are essential components in the management of this condition.

### What is the best therapeutic regimen for patients with IAC?

The management of IAC is largely extrapolated from evidence on candidemia, as no randomized controlled trials have specifically evaluated IAC. Therefore, overall management should follow the same principles applied to candidemia, particularly in patients with extensive or severe disease, emphasizing early initiation of appropriate antifungal therapy with an echinocandin (BII evidence) and effective source control through drainage, debridement, device removal, or surgical intervention whenever feasible (AII evidence). Step-down to oral fluconazole may be considered after 5‒10 days of intravenous therapy in clinically stable patients, if source control has been achieved and antifungal susceptibility has been confirmed (BII evidence). Treatment duration should be individualized, with a minimum of 14-days, and extended in cases of ongoing clinical or radiological signs of infection or when source control has not been achieved (BIII evidence).^4^^,^^40^^,^^198^^,^^199^

In a large multinational cohort study, echinocandins were preferentially prescribed as initial therapy for IAC in patients with greater disease severity or septic shock, although no significant difference in 30-day mortality was observed compared to azole-based regimens.^200^ Critically ill patients often present with altered pharmacokinetics, including increased distribution volume, hypoalbuminemia, impaired perfusion, and drug loss through abdominal drains, which can result in sub therapeutic antifungal exposure, particularly in the early postoperative period.^198^ Moreover, the limited and variable penetration of echinocandins into the peritoneal cavity may reduce their effectiveness and favor the selection of resistant strains.^201^^,^^202^ In this context, some experts have suggested discussing higher-than-standard dosing in patients at increased risk of echinocandin underexposure. However, to date, there is insufficient clinical evidence to support routine dose escalation for all patients.

Rezafungin, a novel echinocandin administered once weekly, has demonstrated comparable efficacy to caspofungin in IC and offers improved tissue penetration in preclinical models, suggesting a role in resistance prevention and lesion-targeted therapy.^203^^,^^204^

Lipid formulations of AmB are a valid empirical or salvage option in critically ill patients, particularly those with sepsis, candidemia, prior antifungal exposure, or infections caused by resistant species, due to their broad-spectrum activity, efficacy against biofilms, and low potential for resistance (AII evidence). Their use may also be considered in clinical scenarios characterized by increased distribution volume, hypoalbuminemia, or impaired perfusion (BII evidence).^4^^,^^205^

Fluconazole, although suitable for treatment, de-escalation or step-down therapy (BII evidence), may require dosing regimen modification and drug monitoring in patients with augmented renal clearance, renal replacement therapy, ECMO, or obesity.^198^ Voriconazole is not recommended as an empirical agent (DIII evidence) but may be considered for targeted therapy in selected cases, provided there is confirmed susceptibility and appropriate pharmacokinetic monitoring (BIII evidence).^206^

Infections associated with continuous ambulatory peritoneal dialysis require immediate catheter removal in addition to systemic antifungal therapy (AII evidence). Recommended treatment options include echinocandins, L-AmB, or fluconazole in patients infected with susceptible strains (BIII evidence).^196^^,^^207^

Table 5 summarizes treatment recommendations for invasive candidiasis involving different body sites.

**Table 5:** Treatment of invasive candidiasis in specific anatomic sites.

Anatomic sites	Proposed treatment (evidence)	Alternative treatment (evidence)	Surgical management and evidence
Intra-abdominal candidiasis	**Echinocandin** (BII)^a^. Step-down to oral **Fluconazole** (BII) once stable.	**Lipid formulation of Amphotericin B** (AII) or **Fluconazole** (BII).	**Source control** (drainage, debridement, or surgery) is essential (AII).
*Candida* endocarditis	**Lipid formulation of AmB** ± **5-FC** (AII) or **Echinocandin** (AII). Step-down to oral **Fluconazole if susceptible (BII)**	**Avoid first line therapy with Fluconazole** (DIII) (it is not active against biofilm)	**Early surgical intervention** (within the first week) for valve infection (BII). Complete **device removal** (BII).
Central nervous system infection	**Liposomal AmB** (3–5 mg/kg) + **5-FC** (BII).	**Fluconazole + %FC and Voriconazole** (BII) if susceptible and L-AMB is not an option.	**Removal of infected shunts**, drains, or infusion devices (BIII).
Osteoarticular infections	**Liposomal AmB** (3–5 mg/kg) or **Echinocandin** (AIII). Step-down to oral **Fluconazole if susceptible**	**Avoid Fluconazole** (CIII) as initial therapy for susceptible strains (it is not active against biofilm)	**Surgical debridement and drainage** are recommended in most cases (AIII).
Ocular infection (endophthalmitis /chorioretinitis)	**Fluconazole** or **Voriconazole** (AIII) for susceptible strains. **Echinocandins (BIII) only for asymptomatic, well-localized chorioretinitis without vitreous involvement.**	**Liposomal AmB** ± **5-FC** (for resistant strains). (BII)	**Pars plana vitrectomy** for chorioretinitis with vitritis (AIII).
Chronic disseminated candidiasis (hepatosplenic)	**Liposomal AmB** or **Echinocandin** (AII). Step-down to oral **Fluconazole if susceptible (BII)**	**Fluconazole** may be an alternative (BII) in stable patients if **AMB** and Echinocandins are not an option.	**N/A** (Therapy may include short courses of **corticosteroids** (CIII) for IRIS-like response).

Note: ^a^ Rezafungin, due to its pharmacokinetic and pharmacodynamic properties, is a promising option for prolonged treatment of deep-seated infections caused by azole-resistant *Candida* species.

### What is the importance of source control in IAC?

Evidence consistently shows that timely and adequate source control is a key determinant of survival in IAC. Pooled analyses and retrospective studies across diverse populations, including critically ill patients, surgical cohorts, and cancer populations, demonstrate that effective source control, when combined with appropriate antifungal therapy, leads to significantly lower mortality. Abscess drainage, debridement, and correction of anatomical defects are critical components.^199^^,^^208^, ^209^, ^210^, ^211^, ^212^, ^213^

In a multinational study of 481 patients, lack of source control was independently associated with a threefold increase in mortality (OR=3.35), with mortality exceeding 60% in patients with septic shock who did not undergo intervention.^208^ In prospective trials, appropriate antifungal therapy combined with procedural source control led to a 67.1% global response rate and 28-day mortality of 24.1%.^199^ In a European ICU cohort, early source control within 72-hours reduced 14-day mortality to 15%, compared to 83% without intervention (HR = 0.23).^209^ Similarly, early antifungal treatment and abscess drainage were protective in IAC, with abscess mortality of 17% versus 40%‒88% in peritonitis cases.^210^

In oncology patients, adequate source control lowered mortality significantly (OR=0.148), although only 44.5% received fully appropriate management.^211^ In elderly patients (>75-years) inadequate abdominal source control is an independent predictor of mortality, alongside end-stage renal disease. In a subgroup analysis of 482 ICU patients, elderly individuals experienced significantly higher mortality (43.5%) compared to non-elderly (20.9%). Those who did not survive were more likely to undergo reoperation, require vasopressors, develop septic shock, and fail to achieve adequate source control within 48h.^212^ Finally, in surgical ICU patients, mortality dropped from 63.2% to 8.8% when both antifungal therapy and source control were implemented within 5-days of diagnosis (combined HR = 0.02).^213^

These findings highlight the critical importance of prompt and aggressive source control in the management of IAC, regardless of patient subgroup or care setting (AII evidence).

### What is the best therapeutic regimen for patients with *Candida* endocarditis?

*Candida endocarditis* may affect native valves, prosthetic valves, or non-valvar endovascular structures, including intracardiac devices such as pacemakers, implantable cardioverter-defibrillators, or ventricular assist devices. Biofilm formation in these settings significantly impacts therapeutic management and often necessitates valve replacement or device removal, along with prolonged antifungal therapy. Mortality remains high, both during hospitalization and at one-year follow-up, with a significant risk of relapse.^40^^,^^214^, ^215^, ^216^, ^217^, ^218^, ^219^, ^220^, ^221^, ^222^, ^223^, ^224^

In a large multicenter cohort study conducted across five tertiary care centers in Brazil between 1980 and 2015, Siciliano et al. (2018) evaluated 78 cases of fungal endocarditis, with *Candida* spp. accounting for 85% of the infections. A total of 66 cases were classified as *Candida* endocarditis. Species identification was achieved in 61 patients, among whom non-*albicans Candida* species predominated (36/61; 59%). Specifically, five cases were attributed to *C. albicans*, while *C. parapsilosis* and *C. tropicalis* accounted for 20 and 13 cases, respectively. The overall in-hospital mortality was 50%. Independent predictors of in-hospital mortality included the presence of acute heart failure and the use of medical therapy without surgical intervention. Conversely, isolated right-sided endocarditis was independently associated with a reduced risk of death.^215^

In the absence of randomized controlled trials defining the optimal regimen for *Candida* endocarditis, initial antifungal therapy may consist of either a lipid formulation of AmB, with or without Flucytosine (5-FC), or an echinocandins (BII evidence).^40^ Both monotherapy and combination regimens have demonstrated favorable outcomes in case series and meta-analyses, including the use of echinocandins, AmB (deoxycholate or lipid formulations), and various combinations.^216^, ^217^, ^218^, ^219^, ^220^, ^221^, ^222^, ^223^, ^224^, ^225^ Although some guidelines and experts have suggested the use of higher-dose echinocandins, robust evidence supporting this strategy remains limited (CIII evidence).^214^^,^^216^ The choice of antifungal agents should, whenever feasible, be guided by the results of in vitro susceptibility testing.^40^^,^^214^

In cases of prosthetic valve endocarditis, the ESCAPE study demonstrated that initial treatment with a L-AmB-based regimen was associated with improved six-month survival compared to echinocandin monotherapy. The addition of 5-FC to L-AmB appeared to further improve outcomes, although statistical power was limited (AII evidence). Importantly, long-term fluconazole maintenance therapy was independently associated with better survival.^218^ In a meta-analysis including 163 rigorously selected cases of *Candida* endocarditis published between 1966 and 2002, adjunctive surgical therapy was associated with a lower mortality compared to antifungal therapy alone, despite considerable heterogeneity. Despite statistical limitations, the findings consistently support the superiority of combined medical and surgical management, especially in patients with left-sided disease or prosthetic valve involvement (BII evidence).^225^

Additional key recommendations include transitioning to oral fluconazole (400‒800 mg/day) in patients with azole-susceptible isolates once clinical stability is achieved and adequate source control has been obtained (BII evidence).^40^^,^^218^^,^^219^^,^^222^ Early surgical intervention, ideally within the first week, is classically recommended for cases involving valvar infection,^40^^,^^214^^,^^219^^,^^221^, ^222^, ^223^, ^224^, ^225^ although some studies have not demonstrated a clear benefit.^218^^,^^220^ Infections involving intracardiac devices require complete device removal.^40^^,^^214^ Although evidence regarding the use of the novel echinocandin rezafungin for *Candida* endocarditis remains limited, a few published cases have demonstrated its safety.^226^^,^^227^ Antifungal therapy should be continued for at least six weeks following surgery and extended in cases of complications. In patients for whom surgery is not feasible, prolonged antifungal therapy is necessary.^40^^,^^214^

### What is the best therapeutic regimen for patients with central nervous system candidiasis?

CNS candidiasis presents with a wide spectrum of manifestations, including brain abscesses, meningitis or meningoencephalitis, vasculitis, ventriculitis, intraventricular fungal balls, hydrocephalus, cranial neuropathies, and stroke syndromes have also been reported in rare cases. CNS involvement typically occurs in the context of disseminated candidiasis or following neurosurgical procedures. Neonates with low birth weight are particularly susceptible to central nervous system involvement secondary to candidemia. *C. albicans* remains frequent in both immunocompromised and immunocompetent hosts, but the prevalence of non-*C. albicans Candida* species has been increasing.^40^^,^^214^^,^^228^, ^229^, ^230^

The optimal treatment regimen for CNS candidiasis remains undefined in the absence of randomized controlled trials. Current recommendations^40^^,^^214^^,^^231^ rely on preclinical models, case series, in vitro susceptibility data, and PK/PD extrapolations.^232^, ^233^, ^234^, ^235^, ^236^, ^237^, ^238^, ^239^, ^240^, ^241^, ^242^, ^243^, ^244^, ^245^, ^246^, ^247^, ^248^, ^249^ Although AmB formulations have limited CSF penetration, they achieve high brain tissue concentrations, particularly at infected sites. L-AmB is preferred due to its superior CNS distribution.^232^, ^233^, ^234^, ^235^ Therefore, L-AmB at a dose of 3‒5 mg/kg daily is regarded as the first-line agent for the treatment of CNS candidiasis, preferably administered in combination with 5-FC at 100‒150 mg/kg/day (BII evidence).^40^^,^^214^^,^^228^

Regarding azoles, fluconazole, despite its excellent CSF penetration, appears less effective than AmB in the first line treatment of *Candida* meningoencephalitis.^244^^,^^245^ It is considered an appropriate step-down therapy, alone or with 5-FC, in clinically improving patients infected with susceptible strains or in those intolerants to AmB, with recommended doses of 400–800 mg/day (6‒12 mg/kg/day) (BII evidence).^40^^,^^214^^,^^228^ Voriconazole, which also achieves therapeutic CSF levels, may be a reasonable alternative in selected cases, particularly for infections caused by fluconazole-resistant species such as *C. glabrata* or *C. krusei* (BII evidence).^40^^,^^214^^,^^246^^,^^247^ Echinocandins, due to limited CNS penetration, are not routinely recommended (DIII evidence).^40^^,^^214^

Removal of infected shunts, drains, or infusion devices is essential for source control and improves therapeutic efficacy (BIII evidence). In cases where device removal is not possible or parenteral therapy fails, intraventricular d-AMB or caspofungin may be used.^40^^,^^214^^,^^228^^,^^248^^,^^249^ The optimal duration of therapy is not well established but generally extends for several weeks and should be individualized based on pathogen, response, and resolution of infection, including normalization of CSF parameters and radiologic improvement.

### How to treat *Candida* osteoarticular infections?

*Candida* osteoarticular infections include arthritis, typically affecting large joints such as the knee and most often resulting from hematogenous spread, osteomyelitis, which occurs in both adults and children and is associated with prior surgery, antibiotic exposure, and immunosuppression, and spondylodiscitis, commonly caused by hematogenous dissemination but occasionally resulting from direct inoculation, particularly after spinal or esophageal procedures.^40^^,^^250^^,^^251^ To date, no randomized clinical trials have been conducted to define the optimal therapeutic approach for these conditions. The following recommendations are therefore derived from case series, retrospective studies, systematic review and meta-analysis, and expert clinical experience.^40^

Initial antifungal therapy with L-AmB (3‒5 mg/kg/day) or an echinocandin is recommended, particularly for critically ill or immunocompromised patients, as well as in infections caused by non-*C. albicans Candida* species or when azole resistance is suspected (AIII evidence). Fluconazole (6‒12 mg/kg/day, typically 400 mg/day) may be used as step-down treatment (AIII evidence). Due to its limited activity against biofilms, fluconazole is not generally preferred as initial therapy for osteoarticular infections (CIII evidence). The total duration of antifungal therapy is generally at least 6-months and should be individualized according to the site and extent of infection, underlying host factors, clinical response, and risk of relapse (AIII evidence). In osteomyelitis and *Candida* arthritis, surgical debridement and drainage are recommended in most cases to ensure source control (AIII evidence). For spondylodiscitis, surgery is indicated in the presence of neurologic compromise or spinal instability.^227^^,^^251^, ^252^, ^253^, ^254^, ^255^, ^256^, ^257^, ^258^, ^259^, ^260^, ^261^, ^262^, ^263^

Evidence from systematic reviews^258^, ^259^, ^260^, ^261^ and a large international multicenter cohort^262^ demonstrates that *Candida* prosthetic joint infections are associated with poor overall outcomes, with cure rates generally ranging from 58%‒85% despite prolonged therapy. *C. albicans* is the predominant pathogen, followed by *C. parapsilosis, C. glabrata,* and *C. tropicalis*, with bacterial coinfection present in up to 50% of cases. Two-stage prosthesis exchange remains the most frequently employed surgical strategy and is consistently associated with superior outcomes compared with debridement, antibiotics, and implant retention, which should be reserved for highly selected patients.^264^^,^^265^ One-stage exchange may achieve suboptimal results comparable to two-stage procedures in selected cases, although supporting evidence remains limited (CIII evidence). Antifungal therapy is typically prolonged (3‒6 months), most often with fluconazole as step-down treatment following initial therapy with L-AmB or an echinocandin, particularly in cases involving resistant isolates. Adjunctive measures, including AmB-loaded cement spacers and, in selected cases, intra-articular antifungal administration, have been reported (CIII evidence). Older age (>70-years), use of debridement, antibiotics, and implant retention, and, in some series, bacterial coinfection are associated with increased risk of treatment failure, whereas infection due to *C. parapsilosis* has been linked to a more favorable prognosis.^258^, ^259^, ^260^, ^261^, ^262^, ^263^

Rezafungin, due to its pharmacokinetic and pharmacodynamic properties, is a promising option for prolonged treatment and for infections caused by azole-resistant species.^227^

### How should ocular candidiasis be treated?

Ocular candidiasis associated with candidemia involves infections of intraocular structures, presenting as chorioretinitis or endophthalmitis. *Candida* endophthalmitis can be exogenous, typically post-traumatic or post-surgical, or more commonly endogenous, resulting from hematogenous dissemination during candidemia. Endogenous forms generally present as isolated chorioretinitis or with vitreous involvement, leading to vitritis. The visual prognosis is closely related to baseline visual acuity and macular involvement at diagnosis.^40^^,^^74^^,^^214^ *C. albicans* is the species most commonly responsible for endogenous endophthalmitis.^40^^,^^214^^,^^266^, ^267^, ^268^, ^269^

No randomized trials have evaluated the optimal treatment for *Candida* chorioretinitis or endophthalmitis. *Candida* endophthalmitis often leads to poor visual outcomes due to suboptimal intraocular drug levels, high fungal burden, or intraocular abscesses.^40^^,^^74^^,^^214^ Achieving adequate drug levels at the infection site, often requiring intravitreal therapy for vitreous involvement, and early pars plana vitrectomy is critical for therapeutic success.^269^, ^270^, ^271^, ^272^, ^273^, ^274^, ^275^, ^276^, ^277^, ^278^, ^279^, ^280^, ^281^ Antifungal choice should consider species susceptibility, with fluconazole or voriconazole preferred for susceptible strains due to excellent ocular penetration.^40^^,^^214^^,^^266^^,^^272^^,^^273^ Voriconazole is frequently used with superior activity against *C. krusei* and *C. glabrata* which are often fluconazole-resistant.^274^ Alternatively, L-AmB demonstrates higher intraocular levels and clinical success in animal models, though human pharmacokinetic data are lacking.^275^, ^276^, ^277^ Intravitreal injections of AmB (deoxycholate or lipid-based) and voriconazole are used in severe *Candida* endophthalmitis to rapidly achieve therapeutic vitreous levels.^276^, ^277^, ^278^

Although echinocandins are first-line agents for CI, their role in ocular candidiasis is limited due to poor intraocular penetration, especially into the vitreous. Experimental and limited human data show therapeutic retinal levels only at supra therapeutic doses.^279^, ^280^, ^281^ Few case reports support echinocandin monotherapy, but most confirmed ocular cases are eventually switched to fluconazole following ophthalmologic assessment. The use of echinocandins for the treatment of candidemia does not seem to have increased the incidence of ocular candidiasis, although some studies have reported an increase in nonspecific ocular findings.^267^^,^^268^^,^^282^

In conclusion, for *Candida* chorioretinitis with vitritis, the preferred regimen for fluconazole/voriconazole-susceptible isolates is fluconazole (loading dose of 800 mg [12 mg/kg], followed by 400‒800 mg [6 mg/kg] daily) or voriconazole (loading dose of 400 mg [6 mg/kg] IV twice daily for 2 doses, followed by 200 mg [4 mg/kg] IV or PO twice daily) (AIII evidence). In cases of asymptomatic, well-localized chorioretinitis (without vitreous involvement), systemic treatment with echinocandins can be considered when candidemia is caused by echinocandin-susceptible *Candida* species (BIII evidence). For azoles resistant strains, L-AmB (3‒5 mg/kg IV daily), with or without 5-FC (25 mg/kg PO four times daily), is recommended (BII evidence). In cases with macular disease or symptoms, add intravitreal d-AmB (5‒10 μg/0.1 mL sterile water) or voriconazole (100 μg/0.1 mL sterile water or saline) (BIII evidence).^214^ For chorioretinitis with vitritis, combine systemic therapy with intravitreal antifungals and consider early pars plana vitrectomy (AIII evidence). Treatment should be continued for ≥ 4‒6 weeks (or 2-weeks in selected asymptomatic cases), guided by lesion resolution on serial ophthalmologic examinations (AIII evidence).

### Chronic disseminated candidiasis

Chronic disseminated candidiasis (CDC), or hepatosplenic candidiasis, is a rare but significant form of invasive candidiasis occurring mainly in patients with hematologic malignancies after prolonged neutropenia, historically linked to AML but increasingly reported in other scenarios.^283^, ^284^, ^285^ CDC should be suspected in patients who develop persistent or recurrent fever after neutrophil recovery, particularly with elevated alkaline phosphatase, and diagnosis relies heavily on imaging, computed tomography or magnetic resonance imaging showing characteristic “bull’s-eye’’ hepatic or splenic lesions, while blood cultures are often negative. Histopathology may show granulomatous inflammation with yeasts or pseudo hyphae, although cultures are frequently sterile, and molecular assays (PCR) may increase sensitivity but lack standardization.

The initial treatment of CDC requires systemic antifungals, with L-AmB or an echinocandin recommended as first-line options (AII evidence). For clinically stable patients who respond to initial therapy, step-down treatment with an azole should be considered (BII evidence). Prolonged therapy (mostly ≥3–6 months) is usually necessary because radiologic resolution is slow, and treatment duration should be individualized based on clinical improvement and radiologic regression of lesions (BIII evidence).^284^, ^285^, ^286^

Because some cases exhibit an immune reconstitution inflammatory syndrome (IRIS)-like inflammatory response, short courses of corticosteroids (0.5 mg/kg/daily, for a period of 2-weeks) may be used to control persistent fever despite adequate antifungal therapy (CIII evidence).^144^^,^^284^^,^^285^

## Prevention

### What are the effective non-pharmacological strategies for preventing candidemia?

Candidemia usually arises from health care exposures, particularly central venous catheters, superimposed on host vulnerabilities. Prevention relies on comprehensive central-line insertion and maintenance protocols, unit-level hygiene measures, minimization of exposures such as unnecessary parenteral nutrition and devices, and rigorous outbreak containment.^2^, ^23^, ^40^

Effective prevention therefore relies on a set of complementary measures. The following sections outline these strategies in sequence, beginning with central-line bundles and extending to unit-wide hygiene, device management, exposure minimization, stewardship, and outbreak control, with special notes for neonatal care and *C. auris*.

#### Central line insertion and maintenance bundles

Key practices include hand hygiene, maximal sterile barriers, and alcohol-chlorhexidine skin prep during insertion, with ultrasound guidance when available. Catheters should be secured with sterile dressings, hubs disinfected at every access, and passive disinfection caps considered. Daily review of line necessity and prompt removal when appropriate remain critical. These measures, when applied consistently, reduce central line-associated bloodstream infection and thereby candidemia rates (AI evidence).^287^^,^^288^

Quality systems including multidisciplinary rounds, data feedback to bedside teams, and leadership accountability, all shown to maintain very low central line-associated bloodstream infection rates over years (BII evidence).^289^

#### Unit-wide skin hygiene

There are no studies supporting the concept that the universal daily bathing with chlorhexidine reduces bloodstream infections by *Candida spp.*, including C. auris.^290^

#### Device and dressing options

When bundle adherence alone does not achieve targets, additional measures include chlorhexidine-impregnated dressings and needleless connector disinfection caps.

Laboratory studies indicate that *C. albicans* can be affected by sub lethal concentrations of chlorhexidine (e.g., induction of apoptosis), suggesting a potential mechanism of action, but without clinical evidence of prevention in patients.^291^ Other *in vitro* trials with impregnated devices (chlorhexidine and chloroxylenol) showed a reduction in colonization by *C. albicans* and other microorganisms, but without a direct correlation with clinical outcomes.^292^

A randomized trial in a pediatric ICU showed that chlorhexidine dressings reduced infections caused by Gram-positive microorganisms, but there was no significant difference in the occurrence of fungal infections, including *Candida* (3.2% in the chlorhexidine gluconate group vs. 4.4% in the control group; p > 0.7).^293^

In conclusion, chlorhexidine-impregnated dressings have been shown to decrease catheter colonization and catheter-related bloodstream infection, predominantly by gram-positive bacteria, but their ability to prevent candidemia remains unproven.

#### Limiting exposures that favor *Candida* translocation is a crucial preventive measure

Early enteral feeding should be prioritized, as late initiation of parenteral nutrition in critically ill patients has been associated with fewer infections overall, including invasive fungal events.^294^ Likewise, removal of urinary and other indwelling devices as soon as they are no longer indicated, and minimizing umbilical or peripherally inserted central catheter dwell time in neonates, are strongly recommended to decrease the risk of catheter-associated *Candida* spp. bloodstream infections (BIII evidence).^294^, ^295^, ^296^

#### Antibacterial stewardship

Although antimicrobial stewardship is “pharmacologic” in a broad sense, its preventive effect on candidemia comes from reducing unnecessary broad-spectrum antibiotics exposition and dysbiosis with *Candida spp*. overgrowth. Antibacterial stewardship is therefore a non-antifungal approach with a potential impact in mitigating candidemia. (BII evidence).^297^^,^^298^

#### *Candida* auris

More than 20 *C. auris* outbreaks have already been reported in Brazil, particularly in the states of Bahia, Pernambuco, Rio Grande do Norte, Minas Gerais, Rio de Janeiro, and São Paulo. In the presence of suspected or confirmed cases, healthcare facilities must immediately implement contact precautions and preventive measures, in accordance with NOTA TÉCNICA GVIMS/GGTES/ANVISA n°02/2022 and subsequent updates.^231^ Institutions should develop and regularly update Standard Operating Procedures (SOPs) for outbreak response, covering patient isolation, environmental decontamination, and staff training. Transmission of *C. auris* occurs primarily through contaminated surfaces and medical equipment in the rooms of colonized or infected patients. Preventive measures should therefore prioritize hand hygiene and rigorous cleaning and disinfection of the environment and shared equipment. Effective disinfectants include hydrogen peroxide, sodium hypochlorite, or other products specifically demonstrated by ANVISA to have activity against *C. auris*. Contact precautions must apply to all healthcare professionals, visitors, and companions for the entire duration of the patient’s stay. Whenever possible, medical devices should be reserved for single-patient use. If exclusive use is not feasible (monitors, ventilators, physiotherapy equipment, or thermometers), these shared items must undergo cleaning and disinfection after every use with hydrogen peroxide or another ANVISA-validated product active against *C. auris*. These procedures align with the approach recommended for other multidrug-resistant organisms but require particularly strict compliance given the persistence of *C. auris* in the healthcare environment (AIII evidence).^231^

The following disinfectants demonstrate high activity against *C. auris*: sodium hypochlorite (≥1,000 ppm; 0.39%–0.65%; 10%), vaporized hydrogen peroxide (8 g/m^3^), peracetic acid combined with hydrogen peroxide <1% (1,200 ppm), and hydrogen peroxide (0.5%‒1.4%). It is important to emphasize that quaternary ammonium compounds show poor activity against *C. auris* and therefore should not be used for environmental disinfection in this context (AIII evidence).^28^, ^299^

### When should antifungal prophylaxis against *Candida* species be given?

#### High-risk abdominal surgical patients

Complicated abdominal surgeries may be associated with an increased risk of IC, including *Candida* peritonitis and candidemia, particularly in critically ill patients. The rationale for antifungal prophylaxis is to decrease the likelihood of such infections and potentially reduce mortality. Fluconazole prophylaxis in this setting has been evaluated in some randomized trials. Eggimann et al. (1999) demonstrated reduced *Candida* peritonitis (4% vs. 35%) and colonization (15% vs. 62%) with 400 mg/day of fluconazole in patients with recurrent GI perforation or anastomotic leaks.^300^ In a larger single-center randomized controlled trial, Pelz et al. (2001) reported a 55% reduction in IC (RR = 0.45; 95% CI 0.21‒0.98) with fluconazole, although without a significant impact on mortality.^301^ Sandven et al. (2002), in a multicenter trial, found that a single intraoperative dose of fluconazole did not significantly reduce mortality (OR=0.21; p = 0.059), though intraoperative yeast isolation was strongly associated with worse outcomes. No benefit was seen in cases of perforated appendicitis, where *Candida* spp. were rarely recovered.^195^ Shorr et al. (2005) analyzed four randomized controlled trials in surgical ICU patients and found fluconazole reduced fungal infections (OR=0.44; p<0.001), but not mortality.^302^

In summary, moderate-quality evidence supports the use of targeted fluconazole prophylaxis in high-risk surgical ICU patients with recurrent gastrointestinal perforation or anastomotic leakage.^300^ In such patients, fluconazole prophylaxis (loading dose of 800 mg followed by 400 mg daily) should be considered (BI evidence). Echinocandins may be an alternative in individuals with prior azole exposure or suspected infection due to azole-resistant *Candida* species, although supporting evidence is limited (CIII evidence). Routine antifungal prophylaxis is not recommended in patients undergoing uncomplicated surgical procedures or in the absence of clearly defined risk factors for invasive candidiasis, owing to concerns regarding antifungal resistance and unnecessary drug exposure (DI evidence).^2^, ^40^

### Hematological malignancies and hematopoietic stem cell recipients

Hematological malignancies and their treatments carry an increased risk of candidemia, particularly during periods of neutropenia and intense mucositis. An approach based on risk stratification has been recommended to guide antifungal prophylaxis against candidemia in patients with hematologic malignancies or undergoing Hematopoietic Stem Cell Transplantation (HSCT).^303^, ^304^, ^305^, ^306^, ^307^

Patients who meet the following criteria are considered at high risk of IC and prophylaxis is recommended for them:^303^, ^304^, ^305^^,^^308^^,^^309^

• Prolonged and profound neutropenia (ANC < 500 cells/μL for > 7-days), as observed during induction or re-induction chemotherapy for acute leukemia or MDS (AI evidence).
• Allogeneic HSCT recipients during the pre-engraftment phase or receiving intensive immunosuppression for graft-versus-host disease (GVHD) (AI evidence).
• Severe mucosal barrier damage due to chemotherapy or conditioning regimens (AII evidence).
• Additional risk factors, such as the use of broad-spectrum antibiotics, CVCs, or parenteral nutrition, may further support the need for prophylaxis in these groups (BII evidence).

More recently, an increasing number of patients with acute myeloid leukemia have been treated with novel targeted therapies, such as hypomethylating agents, midostaurin, or venetoclax in combination with hypomethylating agents.^309^ Antifungal prophylaxis is recommended with moderate strength in most settings and is strongly recommended if the novel acute myeloid leukemia agent is administered in combination with intensive induction chemotherapy (BIII evidence).^303^^,^^304^^,^^310^^,^^311^ Although the risk of candidemia or other fungal infections remains high, these agents have severe concerns regarding drug-to-drug interactions and the need for dose adjustments of the anti-leukemic agent during triazole administration. Venetoclax is metabolized by CYP3A4/5; specific attention must be paid when combined with other drugs that inhibit CYP3A (such as triazoles). For the administration of posaconazole as standard antifungal prophylaxis in AML patients undergoing myelosuppressive remission induction chemotherapy, an evidence-based recommendation for a 75% dose reduction of venetoclax is advised.^303^

In contrast, patients without these high-risk features ‒ such as those undergoing autologous HSCT with rapid engraftment, or those with lymphoid malignancies under low-intensity regimens ‒ typically do not require routine antifungal prophylaxis, unless other host- or treatment-related risk factors are present (CII evidence).^303^

Azoles are considered the first choice for primary antifungal prophylaxis in hematologic patients (AI evidence).^303^ Fluconazole (in a recommended dose of 400 mg daily) is the first choice for anti-*Candida* prophylaxis if the risk for IC is high but the risk for mold infection is low (e.g., autologous HSCT with mucositis). Micafungin has shown efficacy in some settings as an alternative to fluconazole, especially in fluconazole-resistant settings or when fluconazole is contraindicated due to toxicity (BI evidence).^312^, ^313^, ^314^ Trials comparing azoles with a broader antifungal spectrum (posaconazole and voriconazole) to fluconazole have shown no difference in terms of their effect on *Candida* infection but have occasionally shown a trend towards less breakthrough mold disease in settings of high risk for molds (AI evidence). Alternatively, primary antifungal prophylaxis with posaconazole or other mold-active azoles may be recommended in patients with expected long-term neutropenia (i.e., 7-days or longer) from remission-induction chemotherapy for high-risk myelodysplastic syndrome or acute myeloid leukemia, allogeneic HSCT in the pre-engraftment period, or during GVHD in order to prevent mold infections besides candidemia (AI evidence).^310^^,^^315^, ^316^, ^317^ Voriconazole was evaluated during the neutropenic phase of allogeneic HSCT, and it was safe and had efficacy in preventing IFDs (BI evidence).^316^ Isavuconazole may be considered a choice if other azoles are contraindicated due to toxicity, drug-to-drug interaction, or lack of achievement of appropriate serum levels (BII evidence).^311^^,^^318^, ^319^, ^320^ If risk assessment indicates a moderate risk of mold infection, fluconazole combined with active monitoring using serial serum galactomannan is a suitable alternative.^316^ In patients with GVHD, posaconazole is the agent of choice for preventing mold and *Candida* spp. infections (BI evidence).^315^

### Solid organ transplant patients

#### Prophylaxis in liver transplant recipients

Intra-abdominal candidiasis and bloodstream infection are the most frequent invasive diseases, while mold infections, mainly *Aspergillus*, also occur.^321^, ^322^, ^323^, ^324^, ^325^, ^326^ Targeted antifungal prophylaxis in liver transplant recipients is safe and effective for preventing IFDs. Prophylaxis is recommended for patients presenting with at least two of the following risk factors: high MELD scores, re-transplantation, re-exploration surgery, biliary leak, acute renal failure with renal replacement therapy, massive transfusion, choledochojejunostomy, and prior *Candida* colonization. Clinical trials have shown that echinocandins, such as anidulafungin and micafungin, are non-inferior to fluconazole or other standard regimens in high-risk patients. Anidulafungin demonstrated fewer *Aspergillus* colonization and lower rates of breakthrough IFDs compared with fluconazole, while micafungin was associated with similar efficacy, better renal outcomes, and comparable safety. However, concerns persist about echinocandin efficacy in intra-abdominal candidiasis. Overall, fluconazole, anidulafungin, and micafungin are strongly recommended as first-line prophylactic agents against *Candida* in high risk liver transplant recipients (AI evidence).^321^, ^322^, ^323^, ^324^, ^325^, ^326^

### Prophylaxis in lung, heart and heart-lung transplant recipients

After lung transplantation, fungal infections represent 15%–35% of all infectious complications.^327^^,^^328^ *Candida* species are more frequent in the early postoperative period, whereas molds, particularly *Aspergillus*, dominate after the first months. The approach to antifungal prophylaxis differs considerably between centers, but in lung and heart-lung recipients it has traditionally prioritized the prevention of aspergillosis rather than candidiasis.^327^^,^^328^

Evidence for inhaled amphotericin as prophylaxis after lung transplantation is limited and inconsistent, with invasive *Candida* infections occurring despite its use. Systemic prophylaxis with triazoles such as isavuconazole or voriconazole has shown greater efficacy, primarily reducing mold infections and secondarily protecting against candidiasis.^329^ Thus, systemic agents remain the preferred strategy, while inhaled prophylaxis has only a marginal role (BII evidence).

### Prophylaxis in small bowel and pancreas transplant recipients

Recipients of intestinal transplants face one of the highest risks for IFDs due to disruption of the gut barrier, heavy colonization, surgical complications, and intensive immunosuppression.^321^^,^^330^^,^^331^ Current practice favors systemic, risk-adapted prophylaxis, often beginning with a short course of echinocandin followed by an oral azole, while some centers add selective digestive decontamination, though evidence is limited and largely observational. Data remain sparse, mostly from single-center studies, and there is wide variation in prophylaxis strategies across institutions. Long-term azole use requires careful management of drug interactions and, where possible, therapeutic monitoring.^321^^,^^330^^,^^331^

Pancreas transplant recipients with enteric drainage, vascular thrombosis, or post-perfusion pancreatitis are at increased risk for IFDs and should therefore receive antifungal prophylaxis, regardless of whether azoles or echinocandins are used (CIII evidence).^332^^,^^333^

## Candidemia in children

### Which pediatric populations are at highest risk for candidemia in Brazil?

*Candida* bloodstream infections in children remain a significant concern, particularly in neonatal and pediatric ICUs and among patients with underlying comorbidities.^334^

Few studies have specifically evaluated risk factors for candidemia in Brazilian pediatric populations. However, except for neonates, risk factor for invasive candidiasis in children are generally consistent with those documented in adults. These factors include prolonged ICU stay, previous bacterial infections, immunosuppression related to malignancy, its treatment, or transplantation, mechanical ventilation (endotracheal intubation), dialysis, prolonged vancomycin use, recent surgery, use of a CVC, and parenteral nutrition.^335^^,^^336^ Additionally, in pediatric patients**,** primary immunodeficiencies represent an important specific risk factor for the development of candidemia.

In the Neonatal ICU (NICU) setting, major risk factors for the development of candidemia include prematurity, Low Birth Weight (LBW), Very Low Birth Weight (VLBW), immunosuppressive conditions (such as immaturity of the skin and GI tract), prolonged hospitalization, use of TPN, exposure to intensive care devices (mechanical ventilation, central venous catheters), and administration of medications that promote fungal growth, including postnatal corticosteroids and broad-spectrum antibiotics.^337^, ^338^, ^339^, ^340^

A recent study by Silva CM et al., analyzing 44 infants with candidemia, reported that the most affected neonates had prior exposure to TPN and all had received broad-spectrum antibiotics either prophylactically or therapeutically. Most were classified as very low birth weight (VLBW; birth weight between 1000g and 1500g) and had a history of CVC use.^339^ Prematurity and low birth weight were similarly common risk factors in other cohorts.^336^^,^^341^^,^^342^

Another noteworthy recent study by Dassi et al. demonstrated that children with CNS tumors undergoing intensive immunosuppressive therapy, particularly with corticosteroids, were at increased risk for developing candidemia and might benefit from antifungal prophylaxis.^343^

### What is the incidence of candidemia in children in Brazil?

The incidence of candidemia in children in Brazil has been reported in a limited number of multicenter and single-center studies, highlighting the scarcity of pediatric data. The most recent and directly relevant pediatric data from a tertiary hospital in southern Brazil (2016‒2021) found an incidence range of 0.88–1.55 cases per 1,000 hospitalized pediatric patients per year.^344^

Other studies conducted in specific Brazilian hospitals have reported slightly different incidence rates, reflecting variations in patient populations and hospital settings. In a retrospective study involving pediatric patients diagnosed with candidemia between 2014 and 2017, the incidence rate was found to be 1.13 cases per 1,000 patient-days, with a mortality rate of 14%.^345^ This study also emphasized a predominance of infections caused by non-*C. albicans Candida* species *Candida* species. In another important study, Motta et al. evaluated candidemia cases in a pediatric hospital over 10 years and reported an incidence of 3.2 episodes per 1,000 admissions, with non-*C. albicans Candida* species accounting for 59.4% of the infections. The mortality rate associated with candidemia was 50%, emphasizing the severity of these infections in children.^346^

Candidemia remains a significant concern in neonatal ICUs in Brazil, with incidence rates varying by region and patient profile. Studies conducted in different Brazilian states have shown the prevalence of this infection, particularly among Very Low Birth Weight (VLBW) and extremely premature neonates. A study conducted in Pernambuco revealed a prevalence of 10.97% among neonates with suspected septicemia, with *C. parapsilosis* complex and *C. albicans* being the most frequently isolated species.^347^ In a study conducted by Henrique Yuji Watanabe Silva, a total of 1,545 neonates were admitted to the NICU during the study period. Of these, 270 (17%) were Extremely Low Birth Weight (ELBW) neonates, and 1,275 (83%) had a birth weight of ≥1,000g. The study identified 38 cases of IC, with 12 (32%) cases occurring in ELBW neonates and 26 (68%) in those with a birth weight ≥1,000g. The overall incidence of candidiasis was 2.5%, with a higher incidence of 4.4% among ELBW neonates and 2.0% among those with a birth weight ≥ 1,000g.^342^

### Which *Candida* species are responsible for candidemia in children in Brazil, and what are their antifungal resistance patterns?

*Candida* species are significant causes of candidemia in pediatric patients in Brazil, with a notable shift towards non-*C. albicans Candida* species in recent years. Studies have identified several species responsible for bloodstream infections in children, including C. albicans, C. parapsilosis, C. tropicalis, *C. glabrata*, C. haemulonii, and C. guilliermondii.^25^^,^^336^^,^^347^^,^^348^ More detailed data on *Candida* species distribution from the included studies are presented in Table 6.

**Table 6:** Distribution of *Candida* species causing candidemia in Brazilian pediatric clinical settings.

Study (authors)	**Population (age)**	**n**	***Candida* species distribution**
Oliveira et al.^349^	5‒11 years old	104	*C. albicans* 37.5% (n=39)
			*C. tropicalis* 24.0% (n=25)
			*C. parapsilosis* 22.1% (n=23)
			*Pichia anomala* 5.8% (n=6)
			*C. guilliermondii* 4.8% (n=5)
			*C. krusei* 2.9% (n=3)
			*C. glabrata* 1.9% (n=2)
			*C. pararugosa* 1.0% (n=1)
Rodrigues et al.^350^	0‒18 years old	94	*C. parapsilosis* 35.1% (n=33)
			*C. albicans* 28.8% (n=27)
			*C. tropicalis* 23.4% (n=22)
			*C. glabrata* 2.1% (n=2)
			*C. haemulonii* 2.1% (n=2)
			*C. krusei* 2.1% (n=2)
			*C. lusitaniae* 2.1% (n=2)
			*C. guilliermondii* 2.1% (n=2)
			*C. fabianii* 1.1% (n=1)
			*C. kefyr* 1.1% (n=1)
Rodrigues et al.^351^	0‒18 years old	123	*Candida albicans* 20.1% (n=36)
			*C. lusitaniae* 4.0% (n=5)
			*Pichia kudriavzevii* 2.4% (n=3)
			*Wickerhamomyces anomalus* 2.4% (n=3)
			*C. guilliermondii* 1.6% (n=2)
			*C. glabrata* 0.8% (n=1)
			*C. haemulonii* 0.8% (n=1)
			*C. fabianii* 0.8% (n=1)
da Silva et al.^352^	Neonates	44	*C. parapsilosis* complex 38.6% (n=17)
			*C. albicans* 31.8% (n=14)
			*W. anomalus* 11.3% (n=5)
			*C. guilliermondii* 4.5% (n=2)
			*C. glabrata* 4.5% (n=2)
			*C. haemulonii* 4.5% (n=2)
			*C. tropicalis* 2.3% (n=1)
			*C. famata* (current name *Debaryomyces hansenii*) 2.3% (n=1)

In neonatal ICUs, *C. parapsilosis* and *C. albicans* remain the leading species, although resistance concerns arise with isolates such as *C. haemulonii* and *C. grabrata*, which show elevated MICs to azoles and echinocandins. Reports of multidrug-resistant *C. haemulonii* causing neonatal candidemia underscore the need for accurate species identification and advanced diagnostics.^347^^,^^353^^,^^354^

Although no pediatric cases of *C. auris* have been reported in Brazil, infections have been documented in children and neonates in Venezuela and Colombia, emphasizing the potential regional risk. Overall, the epidemiology of pediatric candidemia in Brazil reflects increasing dominance of non-*albicans* species and evolving antifungal resistance, reinforcing the importance of continuous surveillance, timely species identification, and optimized antifungal therapy.^355^^,^^356^

### What is the mortality rate associated with candidemia in children in Brazil?

Candidemia remains a significant cause of morbidity and mortality among pediatric populations in Brazil, with mortality rates varying depending on patient characteristics and care settings.

The first study to systematically investigate mortality risk factors in Brazilian pediatric patients with candidemia was conducted by Pasqualotto et al.,^357^ which analyzed 61 cases, including 14 neonates. The study identified failure to remove the CVC as an independent predictor of early mortality (OR=16.0; 95% CI 2.9–87.8), while late mortality was associated with disease severity measured by PRISM III and PELOD scores.

Among neonatal populations, two key studies highlighted distinct outcomes. In a NICU-based study in São Paulo, Miranda et al.^358^ reported a high crude mortality rate of 45% among neonates with *C. parapsilosis* candidemia ‒ higher than rates observed in Europe and the USA. Factors contributing to this elevated mortality included delayed antifungal therapy and frequent catheter retention. In the study by Silva et al.,^340^ which evaluated 44 neonates with candidemia, the mortality rate of 20.4% was found. Notably, all neonates who did not receive antifungal therapy (6.8%) died, and the use of a CVC was significantly associated with mortality.

In a broader pediatric cohort, Groisman et al.^359^ reported a candidemia mortality rate of 28.3% among 113 children treated at a public hospital in Ribeirão Preto, Brazil, with septic shock emerging as the only significant independent risk factor (adjusted relative risk 2.77; 95% CI 1.12–6.85). Similarly, Motta et al.^360^ found a mortality rate of 32.3% in 65 pediatric patients, identifying mechanical ventilation, dialysis, and acute renal insufficiency as key risk factors.

IFD poses significant morbidity and mortality risks, especially in pediatric patients with neoplastic diseases. In a multicenter retrospective study, Dassi et al.^343^ reported 90 episodes of candidemia in pediatric cancer patients, with a median age of 4.5 years. The 30-day mortality rate was 24.4%, and risk factors for death included older age and ICU admission. Further studies across Brazilian regions revealed mortality rates ranging from 14% to 20.3%.^361^^,^^362^

In summary, pediatric candidemia mortality rates in Brazil range from 14% to over 45%, depending on patient age, clinical setting, underlying conditions and clinical management. Risk factors consistently associated with higher mortality include septic shock, ICU stay, mechanical ventilation, acute renal insufficiency, catheter retention, and delayed antifungal therapy.^363^, ^364^, ^365^, ^366^

### What are the specific considerations regarding the diagnosis of candidemia in children?

Diagnosing candidemia in pediatric patients presents unique challenges, influenced by age-specific clinical features, lower blood volume availability, and limited validation of diagnostic tools in this population. The diagnostic approach can be categorized into microbiological techniques, biomarker-based assays, and molecular methods.

#### Conventional microbiological diagnosis

Standard microbiological diagnostic methods, such as blood culture, microscopy, and histopathology, are the same for adults and children but are limited in sensitivity and timeliness. The sensitivity of blood cultures to detect candidemia ranges between 21% and 71% as reported in autopsy studies^48^ and is believed to be lower for neonates and infants due to sample volume issues. The latest guidelines for the treatment of candidemia, published in 2025, follow the recommendations of the Infectious Diseases Society of America (IDSA) and the American Society for Microbiology (ASM), which state that for infants weighing 1 kg or less, 2 mL should be collected for a single culture, (4% of total blood volume). For those weighing 1.1–2 kg, two samples of 2 mL each can be obtained, (total of 4 mL for culture, 4% of total blood volume). Children weighing 2.1‒12.7 kg should provide 4 mL for the first culture and 2 mL for the second, giving a total of 6 mL, (3% of their blood volume). For patients in the 12.8–36.3 kg range, 10 mL should be collected for each of two cultures, totaling 20 mL, (2.5% of blood volume). Finally, children weighing more than 36.3 kg, should provide 20‒30 mL for each of two cultures, giving a total of 40–60 mL (1.8%–2.7% or less of total blood volume).^40^^,^^367^

Despite these recommendations, blood cultures remain limited by their relatively slow turnaround time, and they may be negative in cases of deep-seated *Candida* infections, such as hepatosplenic candidiasis.

#### Biomarkers and PCR based methods

There are some important differences between children and adults in non-culture-based assays such as BDG testing.

The clinical utility of BDG in children remains controversial due to limited and heterogeneous data. Factors such as prior exposure to intravenous immunoglobulin, albumin, antibiotics, or blood products can lead to false-positive results, and optimal thresholds for positivity have not been clearly established in pediatrics.^368^ Different study designs, study populations, thresholds for positivity, variability in sampling methods or exposure to previous antifungals make results difficult to generalize.

A recent review done by Ferreras Antolin found that the use of BDG in neonates retrieved nine publications, a total of 597 neonates were included, most of them high risk, with 164 proven episodes of Neonatal Invasive Candidiasis (NIC). Only four studies calculated an optimal cut-off for positivity in premature neonates, with values ranging from 99 pg/Ml to 174 pg/mL.^368^ A recent meta-analysis estimated BDG sensitivity and specificity in neonates of 89% (80%‒94%) and 60% (54%‒66%), respectively, when a 80 pg/mL threshold is used in neonates.^369^

A prospective cohort study in children undergoing allogeneic HSCT analyzed 702 serum surveillance samples from 34 patients. The IFD rate for this cohort was 18% (6/34). The authors established an optimal cut-off value of 60–70 pg/mL which yielded a sensitivity ranging between 70% and 100%, a negative predictive value >92%, but the positive predictive value never exceeded 26%.^370^

Despite these studies, BDG continues to be used inconsistently across pediatric centers, with significant variation in access, interpretation, and application (DII evidence).^370^ Further research in large pediatric cohorts at risk for IFD is needed to define optimal cut-off values and clarify the assay’s clinical utility. Due to the limited pediatric evidence, in contrast with adults no definitive recommendations currently support its routine use in children in established guidelines.^40^

PCR-based assays allow for rapid detection with small sample volumes, which is especially advantageous in neonates and infants. The recommendation is similar to adults (DII evidence), reinforcing that the access to non-culture based methods for diagnosing fungi is very limited in most Brazilian centers.^40^^,^^371^

### How to treat candidemia in children?

The general principles for managing IC in pediatric populations mirror those applied to adults, including prompt initiation of antifungal therapy, removal of CVCs when appropriate, control of predisposing factors, and clinical assessment for deep tissue involvement. Identification of *Candida* species and antifungal susceptibility testing are critical components of care in all confirmed cases. Treatment should continue for at least 14-days after clearance of *Candida* from the bloodstream and resolution of neutropenia when present.^40^^,^^372^, ^373^, ^374^, ^375^, ^376^

#### Neonates

Management in neonates requires special consideration due to unique pharmacokinetics and disease manifestations. Based on available data and clinical experience, amphotericin B (AmB), either AmB deoxycholate (d-AmB) or L-AmB or an echinocandin (micafungin or caspofungin) are the first options recommended for the treatment of neonatal candidiasis (AII evidence).^373^ However, in most settings, d-AmB is preferred over lipid formulations in neonatal systemic candidiasis.^377^ The faster elimination observed in neonates is presumably the reason for the reduced nephrotoxicity compared to older children and adults.^378^

Fluconazole, at 12 mg/kg/day with a loading dose of 25 mg/kg, is appropriate in neonates with no prior azole exposure and in institutions with low resistance rates (AII evidence). The literature on the comparative efficacy of fluconazole and amphotericin B in neonatal candidiasis is limited, resulting in a lack of consensus regarding the optimal first-line agent in NICUs.^379^ Fluconazole is especially advantageous for step-down oral therapy due to its excellent CNS and ocular penetration.^372^^,^^374^

Echinocandins such as micafungin and caspofungin are characterized by high-protein binding, liver metabolism, and a wide distribution to tissues, except for the CNS and kidneys. In neonates, these sites are often affected in disseminated candidiasis, and high doses of echinocandins may be required to achieve optimal efficacy. Despite they have shown promising results in neonates, data remain limited. Micafungin demonstrates dose-dependent CNS penetration and has comparable efficacy to amphotericin in randomized trials. Higher doses (up to 10 mg/kg/day) may be necessary to achieve therapeutic levels in CNS infections (BIII evidence).^380^, ^381^, ^382^

Caspofungin appears safe and effective, though it is not approved for neonates in many settings, and anidulafungin the high doses that need to be administered to attain therapeutic CNS levels are associated with Polysorbate 80 (PS80) accumulation and it is approved for children up to 1-month.^40^

#### Children (non-neonatal)

In a multinational observational study from the International Paediatric Fungal Network in children with IC, first-line echinocandin use was associated with reduced crude day-14 failure rates compared to triazoles and amphotericin B (d-AmB, L-AmB, or Amphotericin B Lipid Vomplex ‒ ABLC) (9.8% for echinocandins vs. 13.1% in triazoles/amphotericin group).^383^

In a pediatric study, micafungin was associated with lower mortality (1.9%) and fewer treatment discontinuations (3.8%) compared to L-AmB (mortality 11.1%, treatment discontinuation 16.7%) during treatment of candidemia, whereby mortality at 12-weeks was similar in both arms.^384^

Echinocandins and L-AmB are acceptable first-line treatment options in the pediatric age group, including both neonates (among the echinocandins, micafungin and caspofungin in neonatal period) and older children in accordance with the ECMM/ISHAM/ASM guideline for pediatric patients, both L-AmB and echinocandins (micafungin or caspofungin) are strongly supported as first-line treatments regardless of immunological status or hemodynamics (AII evidence). d-AmB and ABLC have a more limited role due to their nephrotoxic and infusion-related side effect profiles (CII evidence). Fluconazole may be used for step-down therapy once the patient is stable and has a susceptible isolate (BII evidence).^372^

As in neonates, all pediatric patients with candidemia should undergo a thorough search for deep organ involvement (CIII evidence), including eye examinations and CNS evaluation (e.g., lumbar puncture in neonates (AII evidence).^40^^,^^376^

#### Special considerations

• *Candida* urinary tract bezoars: A combination of systemic antifungals (D-AmB or fluconazole) and surgical or interventional urological evaluation is recommended. Echinocandins and lipid-based amphotericin formulations are not effective in this setting due to poor urinary excretion.^40^^,^^372^
• CNS Infections: Optimal therapy in neonates is unclear. Amphotericin B plus 5-FC has demonstrated efficacy in other CNS yeast infections and is considered rational (CIII evidence). High-dose MICA is another emerging option based on pharmacodynamic models (BIII evidence).^40^^,^^372^
• Refractory or persistent IC: In the absence of clinical trials, treatment involves reassessing for adequate source control, switching antifungal classes (e.g., echinocandin to polyene), or considering combination therapy (e.g., polyene plus echinocandin, or either with 5-FC) (CIII evidence).^40^^,^^372^

#### What are the recommended treatment regimens for invasive candidiasis at other sites (including disease involving the CNS) in children?

Candidemia in pediatric patients may be associated with metastatic dissemination to various organs, including the CNS, eyes, kidneys, liver, spleen, and musculoskeletal system. Hematogenous *Candida* meningoencephalitis is a severe and underdiagnosed complication, especially in neonates and immunocompromised children. Management of disseminated candidiasis requires systemic antifungal therapy with agents that exhibit both fungicidal activity and adequate tissue penetration, particularly across the blood-brain barrier.^40^^,^^372^^,^^385^ Table 7, Table 8 shows the recommended dosages of antifungal agents in children with invasive candidiasis, beyond neonatal age.

**Table 7:** Recommended dosages of antifungal agents in children with invasive candidiasis, beyond neonatal age (adapted from^144^).

Invasive candidiasis	Recommended dose	
**Amphotericin B products**		
L-AmB	3 mg/kg qd iv	
ABLC	5 mg/kg qd iv	
d-AmB	1.0 mg/kg qd iv	
**Equinocandins**		
Anidulafungin		
Approved in pediatric subjects >1-month	3 mg/kg qd iv loading dose on d1 and then 1.5 mg/kg qd iv	
Caspofungin	70 mg/m^2^ iv loading dose on d1 and then 50 mg/m^2^ qd iv (max. 70 mg)	
Micafungin	<40 kg: 2‒4 mg/kg qd iv	
	≥40 kg: 100‒200 mg qd iv	
**Azoles**		
Fluconazole	12 mg/kg qd iv (max. 800 mg)	
	12 mg/kg qd orally (max. 800 mg) – switch for oral therapy	
Voriconazole		
Fungistatic. Approved for non-neutropenic patients only. Requires TDM, adequate exposure in the first critical days of treatment is uncertain. Not recommended for initial therapy	Age 2‒11y OR Age 12‒14y and <50kg	9 mg/kg bid iv loading dose on d1 and then 8 mg/kg bid iv and 9 mg/kg bid orally +TDM
	Age ≥15y OR age 12‒14y and >50 kg	6 mg/kg bid iv loading dose on d1 and then 4 mg/kg bid iv and 200 mg bid orally + TDM

ABLC, Amphotericin B Lipid Complex; d-AmB, Amphotericin B deoxycholate; L-AmB, Liposomal Amphotericin B; LD, Loading Dose; TDM, Therapeutic Drug Monitoring; y, Years.

**Table 8:** Recommended dosages of antifungal agents in neonates with invasive candidiasis (adapted from^144^).

Invasive candidiasis		
Drug	Observation	Recommended dose
**Amphotericin B products**		
d-AMB	Fungicidal activity; potential for nephrotoxicity (moderate), infusion related reactions virtually absent in neonates.	0.7‒1.0 mg/kg qd iv
L-AmB	Fungicidal activity; potential for nephrotoxicity (mild). PK in neonates remains undefined, uncertainty regarding optimal dosage.	2.5‒7 mg/kg qd iv
ABLC		5 mg/kg qd iv
**Equinocandins**		
Caspofungin	Fungicidal activity: higher MICs against *C. parapsilosis* group not associated with diminished efficacy. Efficacy in IC comparable to other agents. Dosing designed to approximate drug exposure in adults, appropriate dose to treat HCME unknown.	2 mg/kg qd, equivalent to 25 mg/m^2^ qd
Micafungin	Fungicidal activity: higher MICs against *C. parapsilosis* group not associated with diminished efficacy. Efficacy in IC comparable to other agents. Option for treatment of HCME; dose derived by bridging PK/PD data from animal model of HCME	15 mg/kg qd iv loading dose on d1, then 10 mg/kg qd iv
**Azoles**		
Fluconazole	Fungistatic; inferior outcomes relative to echinocandins not demonstrated in neonates. Efficacy in IC comparable to other agents. Option in patients with no previous azole exposure. Favorable CNS penetration, however, role in HCME not well studied. Studied option for patients on ECMO	25 mg/kg iv loading dose on d1, then 12 mg/kg qd iv
Neonates with documented or suspected hematogenous *Candida* meningoencephalitis		
Micafungin		15 mg/kg qd iv loading dose on d1, then 10 mg/kg qd iv
d-AmB		1 mg/kg qd iv
L-AmB		5–7.5 mg/kg qd iv
d-AmB plus 5-FC		D-AMB:0.7‒1.0 mg/kg qd iv plus 5-FC 100‒150 mg/kg/d iv in 3‒4 divided doses + TDM
Neonates with congenital cutaneous IC		
	Treatment with systemic antifungal agents as recommended for IC	
Neonates with *Candida* bezoar of the urinary tract		
	Treatment with systemic antifungal agents, surgical evaluation, and source control as appropriate	Treatment with both fluconazole and d-AmB achieves adequate drug exposures in the urinary tract whereas administration of lipid formulations of amphotericin B and echinocandins, respectively, do not

ABLC, Amphotericin B Lipid Complex; d-AmB, Amphotericin B deoxycholate; CNS, Central Nervous System; CSF, Cerebrospinal Fluid; ECMO, Extracorporeal Membrane Oxygenation; 5-FC, 5-Flucytosine; HCME, Hematogenous *Candida* Meningoencephalitis; IC, Invasive Candidiasis; L-AmB, Liposomal Amphotericin B; MIC, Minimum Inhibitory Concentration; PD, Pharmacodynamic; PK, Pharmacokinetic.

For children with candidemia and suspected or confirmed CNS involvement, L-AmB at doses of 5‒7.5 mg/kg/day IV is considered the drug of choice due to its superior CNS penetration and safety profile compared to d-AmB, which is used at 1 mg/kg/day IV but carries a higher risk of nephrotoxicity.^40^^,^^214^^,^^235^^,^^386^^,^^387^

Fluconazole at 12 mg/kg/day, preceded by a 25 mg/kg loading dose, may be used in clinically stable patients with susceptible isolates, no prior azole exposure, and in areas with low resistance rates. (BII evidence).^379^ However, azoles may have limited activity in the setting of deep-seated infections and increasing resistance.

Echinocandins, such as micafungin and anidulafungin, are not first-line agents for CNS candidiasis due to limited CSF penetration; however, micafungin has demonstrated therapeutic CNS levels in experimental models and may be considered in selected cases. A pediatric dosing regimen of micafungin 15 mg/kg loading dose, followed by 10 mg/kg/day IV has been suggested in neonates with CNS disease.(BIII evidence).^388^

Anidulafungin may be considered in disseminated candidiasis without CNS involvement, given its proven efficacy and safety in pediatric trials (AII evidence).^389^

Chronic disseminated candidiasis (hepatosplenic candidiasis) is typically seen in children recovering from neutropenia during treatment for hematologic malignancies. Prolonged antifungal therapy is required. Liposomal amphotericin B (3–5 mg/kg/day) or an echinocandin is recommended as initial therapy, particularly during ongoing neutropenia. After hematologic recovery and confirmation of isolate susceptibility, step-down therapy with fluconazole (12 mg/kg/day) may be used until clinical, laboratory, and radiologic resolution (AIII evidence – extrapolated from adult series).^390^

*Candida* endophthalmitis and chorioretinitis are uncommon but potentially devastating complications of candidemia, associated with significant risks of vision loss and long-term morbidity. Diagnosis relies primarily on indirect ophthalmoscopy, the gold standard for retinal visualization. In selected cases, optical coherence tomography or fundus photography may support lesion assessment. Chorioretinitis often responds to systemic antifungals, whereas endophthalmitis may require intravitreal amphotericin B or voriconazole, in addition to systemic therapy. L-AmB and fluconazole are preferred due to their ocular penetration (AIII evidence, derived from the adult literature), while echinocandins are suboptimal in this context.^40^^,^^391^^,^^392^

*Candida* endocarditis, although rare, carries high morbidity and mortality. It usually occurs in patients with prosthetic valves or central catheters. Recommended treatment includes L-AmB (3‒5 mg/kg/day), or a high-dose echinocandin (AII evidence, derived from adults). Surgical intervention is often necessary, and long-term fluconazole suppressive therapy is advised when prosthetic material cannot be removed.^40^

### Antifungal prophylaxis in neonates

IFDs remain a major cause of morbidity and mortality among preterm neonates, particularly those with very low birth weight (VLBW <1,500g) and extremely low birth weight (ELBW < 1,000g). Neonates with abdominal pathology requiring surgery also present an increased risk of Invasive Candidiasis (IC), given that the gastrointestinal tract is the main site of *Candida* colonization.^393^

In Brazil and other Latin American countries, *C. parapsilosis* remains particularly relevant because of its strong association with nosocomial transmission and biofilm formation on indwelling catheters and parenteral nutrition lines. Despite advances in neonatal care, mortality attributable to IFDs in this population remains high, reaching up to 33% in some series, with significant neurodevelopmental sequelae among survivors.^394^ Mortality rates in the NICU are inversely correlated with birth weight, with reported mortality up to 50% in VLBW infants.^395^

The rationale for antifungal prophylaxis is supported by the observation that colonization often precedes invasive disease by several days, providing a window for preventive intervention. Clinical signs of candidemia in neonates are nonspecific, overlapping with bacterial sepsis, and delays in antifungal initiation markedly increase mortality.^396^

Fluconazole remains the most extensively studied agent for neonatal prophylaxis. At birth, the majority of VLBW neonates are not yet colonized or have low-density colonization, creating a unique opportunity for antifungal prophylaxis to prevent or limit colonization. Without prophylaxis, up to 60% of VLBW neonates become colonized with *Candida* by the third week of life.^396^ A recent systematic review and meta-analysis by Anaraki et al. reported a significant reduction in *Candida* colonization rates among VLBW neonates receiving fluconazole prophylaxis.^397^

Several studies have indicated a significant reduction in the incidence of colonization in vulnerable neonatal populations following the administration of fluconazole prophylaxis.^398^ A prospective multicenter point-prevalence study across 27 European NICUs revealed that fluconazole was the most frequently used antifungal for prophylaxis, employed in over 98% of cases.^399^ The optimal regimen varies, but the most recent guidelines recommend fluconazole 3–6 mg/kg twice weekly for 6-weeks in institutions with a high IC incidence (>10%) and in neonates with birth weight < 1,000g (AI evidence) Prophylaxis is strongly advised for extremely low birth weight infants in centers with IC incidence above 2% and low rates of fluconazole resistance.^40^^,^^400^ Although fluconazole prophylaxis is widely used, concerns persist regarding potential resistance development. A secondary analysis of a randomized controlled trial showed that, while fluconazole exposure increased the median MIC among colonizing *Candida* isolates, values remained within the susceptible range (median MIC = 1 μg/mL), and resistance was rare. Similarly, large multicenter surveillance studies have not demonstrated significant fluconazole resistance emergence in NICUs using prophylaxis.^401^^,^^402^ Overall, evidence from randomized trials, meta-analyses, and updated guidelines supports the efficacy and safety of fluconazole prophylaxis in high-risk neonates.

## Access policies for antifungal medications in Brazil

Brazil has established a national framework for the surveillance and management of invasive and/or endemic mycoses through an executive group within the Ministry of Health (MoH), supported by the Technical Advisory Committee on Endemic and Opportunistic Mycoses. This structure coordinates epidemiological monitoring and ensures nationwide access to antifungal therapy.

The MoH recommends anidulafungin as first-line therapy for microbiologically documented candidemia and other invasive *Candida* infections. In specific clinical scenarios where echinocandins are not appropriate, such as central nervous system infection, endophthalmitis, or endocarditis, the lipid formulations of amphotericin B or voriconazole may be used. Access to antifungal agents is centrally regulated and contingent upon confirmed diagnosis, including positive blood cultures, isolation from sterile sites, and/or histopathological evidence.

The incorporation of echinocandins into the public health system was formalized by Portaria SCTIE/MS n° 55 (July 26, 2022), with subsequent operational guidance detailed in Nota Técnica n° 7/2024-CGTM/DATHI/SVSA/MS.

## Final considerations

This guideline underscores that candidemia and invasive candidiasis remain major causes of morbidity and mortality in Brazil, with mortality rates that have not substantially improved over the past decades despite the expansion of diagnostic tools and antifungal therapies. The persistent excess mortality, particularly in critically ill populations, reflects delays in diagnosis, restricted access to first-line antifungal agents, incomplete source control, and heterogeneity in clinical practice across institutions. By integrating national epidemiologic data with international evidence, these recommendations seek to narrow the gap between best evidence and bedside practice, emphasizing early recognition, timely initiation of appropriate antifungal therapy, and systematic implementation of bundled care measures.

A central contribution of this consensus is the contextualization of global guidance to the Brazilian healthcare setting. The document highlights structural constraints, such as limited availability of fungal biomarkers, uneven access to echinocandins, and restricted deployment of advanced microbiological tools, while providing pragmatic algorithms that prioritize feasibility without compromising quality of care. The emphasis on the clinical monitorization of high-risk patients, standardized diagnostic pathways, early initiation of the adequate antifungal regimen and targeted application of echocardiography and ophthalmologic evaluation aims to optimize resource use and reduce unwarranted practice variation. Furthermore, the reinforcement of antifungal stewardship principles, including de-escalation strategies and susceptibility-guided therapy, aligns clinical effectiveness with sustainability.

Finally, this guideline affirms that improvement in outcomes will depend not only on pharmacologic advances but also on system-level interventions, including strengthening laboratory capacity, expanding antifungal access, and ensuring early infectious diseases consultation as a standard of care. Future research priorities include population-based surveillance, outcomes research in special populations, development of new sensitive and specific biomarkers to early detect episodes of invasive candidiasis and translational studies to refine pharmacokinetic–pharmacodynamic targets in critically ill patients. Ultimately, this consensus is intended as a dynamic framework, subject to periodic updates, that supports clinicians, informs policymakers, and fosters coordinated national strategies to reduce the burden of candidemia and invasive candidiasis in Brazil.

## Author’s contribution

ALC and ACP: Conceptualization, study design, data curation, formal analysis, writing of the original draft, supervision, and validation of the final manuscript. MMCM, FC, DRF, MG, DWCLS, and FQT: Contribution to specific sections of the manuscript, data curation, critical revision of the text, and validation of the final version.

## Funding

The authors were contracted by Mundipharma and Sandoz to develop educational materials related to the consensus text. Of note, the sponsors had no access or any influence on the content of the Consensus along the writing of the manuscript.

## Data availability statement

The data that support the findings of this study are available from the corresponding author upon reasonable request.

## Conflicts of interest

ALC has received support for educational activities from Knight-United Medical, Sandoz, Mundipharma, Accord, Gilead, IMMY, and research grant from Bruker, Knight, Centers for Disease Control-USA (Award Number 1 NU3HCK000010-01-00) and São Paulo Research Foundation ‒ FAPESP (Grants 21/10599-3). MMCM has received support for attending educational meetings from Knight and Mundipharma. FC reports honoraria for lectures from Pfizer, Knight, and Sandoz, and participation in advisory boards for Knight and Pfizer. DRF has received research grants and consulting fees from Pfizer, Merck, and Gilead Sciences; participation on Data Safety Monitoring or Advisory Boards for Gilead Sciences, Merck, and GlaxoSmithKline (GSK); has given paid lectures on behalf of Mundipharma, Sandoz, Pfizer, GSK, Merck, Gilead Sciences, Knight Pharmaceuticals; and received nonfinancial research support from IMMY. ACP has received research grant support from Gilead Sciences, MiraVista Diagnostics, IMMY, the Pan American Health Organization, the Brazilian Innovation Agency – FINEP, and the NIH (Award Number 1R01AI195133-01). He has consulted for Gilead Sciences and Elion Therapeutics, and received support for educational activities from Gilead Sciences, IMMY, Sandoz, Mundipharma, bioMérieux, MSD, Europharma, Pfizer, Takeda, Sanofi, Knight Therapeutics, GSK, and Astellas. Other authors have no conflict of interest to declare.
